# The impact and mechanisms of educational human capital on the risk of relapse into poverty: Evidence from Chinese micro-families

**DOI:** 10.1371/journal.pone.0357459

**Published:** 2026-09-10

**Authors:** Siliang Guo, Yan Qu, Youjia Liu

**Affiliations:** 1 School of Economics and Management, Qilu Normal University, Jinan, China; 2 Research Center of the China-Pakistan Economic Corridor, Kashi University, Kashi, China; University of Salento: Universita del Salento, ITALY

## Abstract

Educational human capital, as a critical driver of social development, plays an instrumental role in alleviating poverty and mitigating the risk of its recurrence. Drawing on human capital theory and utilizing data from four waves of the China Family Panel Studies (CFPS) spanning 2014–2020, this paper empirically examines the impact of educational human capital on the risk of relapse into poverty and explores its underlying mechanisms. The analysis employs panel data fixed-effects models, System GMM, and quantile regression. The findings indicate that: (1) Educational human capital significantly reduces the likelihood of household poverty relapse. (2) Mediation analysis reveals that this protective effect operates through three primary pathways: promoting non-agricultural employment among family members, increasing household income, and improving family health status. (3) Moderation analysis demonstrates that household investment in education substantially enhances the efficacy of educational human capital in mitigating relapse risk, whereas household health expenditure tends to attenuate this effect. (4) Heterogeneity analysis suggests that the marginal impact of educational human capital intensifies as the risk of poverty relapse rises, with the effect being more pronounced in eastern regions and urban areas. These findings deepen the understanding of how educational attainment influences poverty dynamics, offering empirical evidence and policy insights for developing countries aiming to consolidate poverty alleviation achievements and prevent poverty recurrence through educational strategies.

## 1. Introduction

While the eradication of absolute poverty marks a pivotal milestone in global development, the focus of anti-poverty strategies has shifted from short-term alleviation to the sustainability of these outcomes. In China, despite the historic achievement of lifting nearly 100 million rural residents out of poverty by 2020, the risk of poverty relapse remains a critical challenge. Policymakers now confront the dual mandate of consolidating gains and addressing structural vulnerabilities. The 2022 and 2023 “No. 1 Central Documents” explicitly emphasize the urgency of preventing large-scale poverty resurgence, noting that millions of previously impoverished individuals remain vulnerable to economic shocks and environmental changes [1,2]. Therefore, constructing robust mechanisms to prevent poverty relapse has become the core objective of the current developmental phase.

Existing research on the risk of poverty relapse predominantly centers on measurement methodologies and the identification of determinant factors, leading to the development of multidimensional evaluation indices that span natural, economic, and social domains [3,4]. These influencing factors are generally categorized into external shocks, such as natural disasters and policy withdrawal, and internal vulnerabilities, including health status and income instability [5,6]. Amidst these dynamics, education is widely recognized as a fundamental driver for blocking the intergenerational transmission of poverty. Grounded in human capital theory, education enhances cognitive abilities and labor productivity, thereby fostering the economic resilience required to mitigate these relapse risks [7,8].

However, despite the consensus on the importance of education, a critical theoretical and empirical gap persists. The current literature focuses primarily on the direct impact of education on poverty alleviation. However, empirical validation of the mediator pathway is still limited. Specifically, the “black box” mechanisms through which educational human capital inhibits the structural dynamics of poverty relapse are largely unexplored. It remains unclear how education interacts with intermediate variables such as non-agricultural employment stability, health capital accumulation, and income structure. Consequently, the extent to which these interactions buffer households against relapse risk in a post-poverty context is not fully understood. This lack of mechanistic insight constrains the ability to formulate precision-oriented policies.

To address this gap, this study empirically investigates the impact of educational human capital on the risk of relapse into poverty and its underlying mechanisms. Using data from the 2014–2020 China Family Panel Studies (CFPS), we employ panel fixed-effect and quantile regression models to rigorously test these relationships. The marginal contribution of this study to the existing literature is threefold: First, unlike studies focusing solely on direct effects, this paper contributes an in-depth mechanism analysis. We empirically validated the mediating pathways for non-farm payroll employment, household income, and health status. This provides a granular understanding of how education functions as a buffer against the risk of relapse into poverty, providing a scientific basis for interventions that go beyond simple tuition subsidies. Second, regarding methodology, we construct a comprehensive risk assessment index system for poverty relapse risk. By integrating the five domains of material conditions, financial resilience, social capital, human capital and existential risk, we provide a more holistic measurement perspective than traditional single-dimensional income indicators, allowing for a more accurate capture of latent poverty risk. Third, we provide a nuanced analysis of heterogeneity. By examining how the impact of education varies across regions and household categories, this study informs the formulation of geographically tailored strategies to address the one-size-fits-all limitations of previous studies and to optimize policy resource allocation.

The organizational structure of this study proceeds as follows: Section 2 conducts a comprehensive literature review to establish theoretical foundations, while Section 3 develops the analytical framework and formulates empirically testable hypotheses. Section 4 details the methodology design, including model specification and variable operationalization rationales. Section 5 presents the empirical findings through baseline regression analyses, causal mechanism verification, robustness evaluations, and heterogeneity examinations. Section 6 translates empirical results into policy implications, offering actionable recommendations for poverty alleviation strategies. Finally, Section 7 concludes by synthesizing principal contributions and proposing directions for subsequent research expansion.

## 2. Literature review

### 2.1 Related research on relapse into poverty

The conceptualization and dimensionality of poverty relapse risk within academic discourse goes beyond mere economic indicators, encompassing both the vulnerability of household income streams and their susceptibility to exogenous shocks such as natural disasters, macroeconomic fluctuations, and systemic disruptions. Scholars increasingly emphasize the necessity of multi-pronged institutional frameworks to forestall poverty resurgence, particularly by strengthening healthcare infrastructure, elevating educational attainment, and enhancing labor market resilience [9]. Building on this theoretical foundation, the present study defines the risk of relapse into poverty as the latent likelihood that an individual or household will fall into or return to poverty due to the compounding effects of structural vulnerabilities, income instability, and insufficient capacity for adaptation. This definition integrates both economic and non-economic dimensions, recognizing that the resurgence of poverty often stems from the interplay of inadequate risk mitigation mechanisms, limited human capital accumulation, and exposure to multidimensional stressors. This phenomenon is inherently uncertain and dynamic, manifesting itself as either a return to poverty for the previously poor or a descent into poverty for the non-poor. These risks arise from a variety of sources, ranging from external shocks – such as natural disasters, disease, policy withdrawal and market volatility – to internal factors, including personal incapacity and resource scarcity.

Current scholarly inquiry into poverty relapse risk measurement has crystallized a multidimensional theoretical architecture, characterized by four principal research paradigms. The foundational paradigm originated with the United Nations Development Programme’s Multidimensional Poverty Index, which operationalized poverty assessment through three core dimensions—health, education, and living standards—via ten measurable indicators. Alkire et al.[10] subsequent refinement through the AF dual-threshold methodology systematized identification and aggregation processes, establishing a methodological cornerstone for multidimensional poverty measurement with demonstrated explanatory power in developing economies. Empirical adaptations have further enriched this framework; Angulo et al. [11] innovatively incorporated employment as a fourth dimension in the Colombian case study, constructing an education-health-employment-living standards model that captures the structural poverty dynamics in developing contexts. Beyond traditional welfare metrics, emerging scholarship adopts capital endowment perspectives, exemplified by Du et al. [12] four-dimensional capital framework (natural, financial, physical, and human), which shifts analytical focus from static poverty conditions to capital deprivation mechanisms, thereby enabling dynamic risk monitoring. Most recently, Ma et al. [13] advanced a sustainability-oriented paradigm integrating environmental carrying capacity, social support systems, and economic resilience, aligning poverty alleviation with Sustainable Development Goals through a complex systems lens. This evolutionary trajectory reveals three transformative trends: the expansion from basic needs assessment to developmental capability evaluation, the transition from cross-sectional analysis to processual monitoring, and the convergence of disciplinary paradigms into interdisciplinary syntheses, collectively redefining poverty relapse risk research as a nexus of multidimensional metrics, dynamic capital flows, and socioecological resilience.

### 2.2 Related research on educational human capital

Within poverty alleviation research, education’s pivotal role in human capital development has garnered significant scholarly attention. Serving as the primary mechanism for transmitting knowledge, technical competencies, and adaptive capacities, educational systems constitute the most efficient pathway for human capital enhancement. This transformative process elevates labor force quality while activating endogenous development dynamics among impoverished populations, generating sustained income growth that reinforces poverty prevention infrastructure. Beyond economic dimensions, education-driven human capital accumulation produces profound socio-structural benefits by fostering inclusive social participation. Equitable educational opportunities cultivate individual self-efficacy, mitigate marginalization risks for vulnerable demographics, and strengthen communal cohesion through enhanced socio-cultural integration. Such multi-layered empowerment effectively disrupts intergenerational poverty transmission by addressing systemic disadvantages across political participation, economic opportunity, social capital formation, and welfare access [14]. Vocational education frameworks warrant particular emphasis as critical components of human capital strategies, with targeted skill development initiatives demonstrating measurable impacts on employment quality and income equity. By aligning workforce competencies with evolving market demands, these interventions contribute to narrowing urban-rural economic disparities while establishing durable anti-poverty mechanisms [15]. Furthermore, education’s salutogenic effects extend to health capital formation, as elevated educational attainment correlates with improved socioeconomic status, enhanced health literacy, and reduced psychosocial vulnerabilities [16]. Collectively, these educational interventions create compounding poverty-reduction effects by concurrently elevating human capital endowments, economic resilience, and physiological well-being. This multi-systemic enhancement not only strengthens individual agency but also establishes communal safeguards against poverty resurgence through inclusive development paradigms.

### 2.3 Research on the relationship between educational human capital and the risk of relapse into poverty

The link between human capital accumulation and poverty dynamics has been extensively examined in development economics. The consensus, grounded in classical human capital theory, identifies education as a fundamental driver of labor productivity and earning potential. International evidence from both developed and developing economies consistently demonstrates that educational attainment is negatively correlated with poverty incidence [17]. For low-income households, education functions as a critical asset for upward mobility, enabling individuals to transcend socioeconomic barriers and break the intergenerational transmission of poverty. Specifically, households with higher human capital stocks exhibit stronger resilience against external shocks, thereby reducing the likelihood of falling back into destitution [18].

However, the relationship between education and poverty alleviation is neither linear nor universally guaranteed. From an international comparative perspective, the impact of education varies significantly across contexts. In developed economies, the challenge often lies in the skills mismatch, whereas in many developing nations in Sub-Saharan Africa and Latin America, the supply-side expansion of education has not always been met with sufficient demand-side job creation, leading to the phenomenon of educated unemployment [19].

This complexity is particularly pronounced in the Chinese context, where the rapid expansion of education coexists with structural rigidity. While education generally inhibits poverty, scholars caution that, under certain conditions, educational investment can paradoxically exacerbate vulnerability. This counterintuitive phenomenon, often referred to as education-induced poverty, can be explained through three theoretical lenses.

First, the crowding-out effect of educational costs. For marginalized families, the direct and opportunity costs of education are substantial. High educational expenditures can crowd out other essential consumption or productive investments, pushing households below the poverty line before the long-term returns of education materialize [20]. This is in contrast to the Western context where student loans are prevalent; in rural China, the liquidity constraint is a more immediate trigger for poverty relapse [21].

Second, the screening hypothesis and credential inflation. Education may serve only as a signal, not as a productivity enhancer. In a labor market characterized by rapid educational expansion, such as China’s policy of following higher education expansion, the supply of graduates may exceed the availability of high-quality jobs. This leads to credential inflation, where the marginal return to lower levels of education diminishes [22]. As a result, individuals from poor families who invest in low-quality or unmarketable education may find themselves stuck in the low-wage sector and fail to recoup their investment.

Third, factor allocation and diminishing returns. Following the law of diminishing marginal returns, the efficacy of educational capital depends on complementary factors such as infrastructure, market access and social networks. In remote, poverty-stricken areas, the lack of these complementary inputs limits the economic returns to education, potentially leading to a low-level equilibrium trap [23].

Hence, preventing a relapse into poverty requires more than simply increasing school attendance. It requires a nuanced understanding of how educational quality, cost burdens and labor market integration interact. While education remains a cornerstone of sustainable livelihoods, its potential to foster shared prosperity in China depends on mitigating these structural risks and ensuring that human capital accumulation is effectively translated into stable income streams.

In summary, while the existing research on the impact of educational human capital on the relapse of poverty provides a solid foundation for this study, several critical gaps remain. First, in the current phase of relative poverty governance, research remains inadequate. Previous studies have focused primarily on income levels, often overlooking the holistic livelihoods of impoverished families. Defining poverty by economic indicators alone is no longer sufficient; There is a pressing need to adopt a multidimensional perspective to analyze the complex features of poverty. Second, the literature has focused on measuring the risk of relapse and identifying the influencing factors. However, the analysis of the underlying mechanisms is relatively weak, and quantitative studies exploring these causal pathways are notably scarce. Third, existing research tends to seek general principles, often neglecting significant heterogeneity. It often fails to account for regional disparities in geographic and economic development, as well as household distinctions such as demographic structure and urban-rural attributes. This oversight limits the practical applicability of the findings, making it difficult to tailor policies to the specific needs of diverse regions and households.

## 3. Theoretical analysis and research hypothesis

### 3.1 Educational human capital and the risk of relapse into poverty

Education plays a critical role in mitigating the risk of relapse into poverty by fostering sustainable socioeconomic progress. Classical human capital theory posits that higher educational attainment enhances labor productivity and income levels, thereby acting as a fundamental buffer against economic shocks [2]. However, in the specific context of China, the poverty alleviation function of education must be understood within the framework of the country’s unique institutional constraints, particularly the urban-rural dual structure and regional development disparities.

First, education serves as a critical mechanism to overcome the institutional barriers imposed by the urban-rural dual structure. Historically, this structure has segregated resource allocation, leaving rural households with limited access to social security and public services, thereby increasing their vulnerability to relapse into poverty. Educational accumulation empowers rural individuals to transcend these institutional boundaries. By acquiring higher credentials, rural laborers can access formal employment sectors that offer social insurance and stability, effectively reducing the precariousness associated with the informal rural economy [24].

Second, education mitigates the adverse effects of regional development imbalances. In China’s vast geography, economic opportunities are spatially concentrated in the eastern coastal regions and urban centers. For households in less developed Central and Western regions, education acts as a “spatial bridge,” enhancing their capability to migrate and integrate into more developed regional markets. It reduces the costs of migration and information asymmetry, allowing households to diversify their income sources beyond local, often fragile, economic ecosystems. By breaking the intergenerational transmission of poverty through these institutional and spatial channels, education enhances the long-term stability of poverty alleviation efforts [12]. Consequently, human capital not only raises immediate income but also structurally lowers the risk of relapse by integrating households into broader, more resilient economic networks [25]. Based on this analysis, we propose the following hypothesis.

H1: Educational human capital has a significant inhibitory effect on the risk of relapse into poverty.

### 3.2 Mechanism analysis of the impact of educational human capital on the risk of relapse into poverty

Schultz stressed that education enhances workers’ ability to distribute. Specifically, their capacity to perceive and respond to economic opportunities. In the Chinese context, this allocation mechanism is heavily influenced by the labor market segmentation resulting from the urban-rural dual structure. For households recently lifted out of poverty, the risk of relapse is often tied to their dependence on the low-productivity agricultural sector, which is highly vulnerable to natural disasters and price volatility.

Education facilitates the transition from agricultural to non-agricultural employment and acts as a signalling device to break down labour market fragmentation. In a dual economy, the labor market is often divided into a primary sector (high wage, stable, often urban) and a secondary sector (low wage, unstable). Without adequate human capital, rural laborers are often confined to the secondary sector, even when they leave agriculture. Higher educational attainment enables rural laborers to overcome statistical discrimination and entry barriers, facilitating access to higher-value non-agricultural jobs [6,26].

Moreover, this career shift is crucial for income diversification and risk hedging. Research has shown that better-educated household members demonstrate a significantly higher propensity to transition into non-farm employment. This mobility yields two distinct benefits: immediate income gains via wage premiums in the industrial and service sectors, and an enhanced capacity to absorb economic shocks through diversified income streams [27]. By shifting the allocation of labour from subsistence farming to the modern sector, households can effectively decouple their livelihoods from agricultural risks. Empirical studies confirm that increased non-farm labor participation, driven by educational upgrades, contributes significantly to aggregate household income and provides the foundational economic security needed to prevent a return to poverty [6,26,28]. Based on the preceding analysis, we propose the following hypothesis.

H2: Educating human capital can reduce the risk of relapse into poverty by increasing the non-farm employment rate of household members.

Grounded in the wage-productivity nexus of human capital theory, education is directly correlated with higher marginal productivity and, consequently, higher earnings. Stable and elevated household income functions as the most immediate financial buffer against economic shocks. By enabling individuals to secure sustainable livelihoods, education ensures that income growth outpaces consumption volatility, thereby cementing poverty alleviation outcomes. Moreover, household income significantly modulates the effectiveness of education in poverty reduction. On the one hand, educational advancement is intrinsically linked to personal income growth. Higher educational attainment among family members increases the probability of securing high-wage employment, which raises aggregate household income and enhances the long-term stability of poverty alleviation efforts [29]. Elevating household income amplifies the efficacy of education, serving as a critical pathway for realizing the benefits of human capital, particularly among low-income groups [30]. Higher-income households, on the other hand, have greater capacity to invest in their children’s education, fostering a virtuous cycle that sustains poverty alleviation across generations. However, it is important to note that income inequality can exacerbate educational disparities, impeding the transmission of achievement and the likelihood of escaping poverty [12]. In addition, beyond monetary gains, education significantly reduces multidimensional poverty by enhancing labor quality and market competitiveness, thereby expanding employment opportunities and optimizing resource utilization. Consequently, increasing household income represents a vital mechanism for enhancing the quality of education and promoting sustainable poverty alleviation. Based on the preceding analysis, we propose the following hypothesis.

H3: Educational human capital endowments can reduce the risk of relapse into poverty by raising household income.

Education facilitates the accumulation of health capital as educated individuals are more inclined to adopt healthy behaviors, efficiently utilize medical resources, and maintain superior health status. Given that robust health is a prerequisite for sustained labor force participation, education indirectly safeguards household economic stability by minimizing the risk of disease-induced poverty-a primary driver of poverty relapse. Empirical investigations have consistently demonstrated a significant positive correlation between educational attainment and health outcomes. Individuals with higher educational qualifications showed marked improvements in preventive care and chronic disease management. These health-enhancing effects translate directly into reduced household health-care expenditures, thereby strengthening household financial resilience [31]. Research has shown that parental education significantly affects pediatric health trajectories, with better educated caregivers demonstrating superior capacity to manage chronic conditions and adopt health-promoting behaviors [30]. In addition, educated households consistently report better access to health infrastructure, sanitation and clean energy, along with improved mental well-being and lower prevalence of harmful habits such as tobacco use, all of which contribute to elevated household health status [8]. Functioning as a critical determinant of health literacy and resource mobilization, education empowers low-income families to effectively navigate health risks, adopt preventive lifestyles, and mitigate disease susceptibility [32]. Longitudinal evidence from Hubbard [33] reinforces this nexus, showing that households with higher educational attainment maintain superior health profiles over time, resulting in reduced health care cost burdens and enhanced long-term financial stability. Collectively, these findings suggest that educational investment generates a compound health dividend that reinforces poverty alleviation outcomes through multiple interconnected pathways. Based on the preceding analysis, we propose the following hypothesis.

H4: Educating human capital endowments reduces the risk of relapse into poverty by improving the health status of family members.

### 3.3 Analysis of the moderating effect of educational human capital on the risk of relapse into poverty

Familial educational investment significantly amplifies the mitigating influence of human capital endowments on the risk of poverty relapse. Grounded in the Human Capital Theory, which conceptualizes education as a high-return investment essential to enhancing future productivity, this hypothesis suggests that financial resources allocated to learning are critical to building economic resilience. First, increased financial allocation to home-schooling accelerates human capital accumulation, thereby stabilizing long-term poverty reduction outcomes. Empirical evidence corroborates this, showing that education spending has an intergenerational anti-poverty effect. Specifically, parental investment in learning resources substantially improves offspring educational outcomes and strengthens the economic mobility function of human capital [34]. This dynamic is further elucidated by the theory of diminishing marginal utility, which explains why educational expenditure exhibits a disproportionate anti-poverty efficacy among agrarian and economically disadvantaged households. For these groups, often facing liquidity constraints, marginal increases in educational investment yield significantly more pronounced reductions in poverty vulnerability compared to affluent households [35]. Second, viewed through the lens of the education production function, strategic home-based educational investments serve as critical inputs that directly elevate learning quality, which in turn correlates with sustained household income growth trajectories [36]. However, this positivity relation operates within structural constraints. Consistent with the theory of intergenerational transmission of inequality, household income disparities significantly modulate access to education. As a result, widening economic gaps may exacerbate educational disparities, reinforce the mechanisms of inequality, and potentially attenuate the poverty alleviation effects of human capital accumulation. Based on the above analysis, we propose the following hypothesis.

H5: Family education investment positively promotes the weakening effect of educational human capital endowment on the risk of relapse into poverty.

Excessive health spending has a detrimental crowding-out effect on investment in education, thus dampening the contribution of human capital to poverty alleviation. Theoretically anchored in the framework of household budget constraints and intertemporal choice, this phenomenon arises from the intense competition between immediate necessities for survival and long-term developmental investments. Given that demand for health care is often inelastic and urgent, high health spending creates acute financial pressures, forcing households to reallocate resources away from human capital accumulation. In the context of China’s rural revitalization, this trade-off is particularly pronounced due to the “liquidity constraints” faced by rural households. When health shocks occur, the immediate need to preserve health capital cannibalizes the financial resources required for educational human capital, effectively inhibiting the latter’s stabilizing role in household economics [12,37].

Deepening the analysis within the Chinese institutional context, the urban-rural dual structure significantly exacerbates this vulnerability. Although the New Rural Cooperative Medical Scheme has achieved broad coverage, effective reimbursement rates for critical illness and outpatient services in rural areas often lag behind urban employee insurance schemes. In addition, uneven distribution of high-quality medical resources forces rural residents to seek care in upper-tier hospitals, incurring higher out-of-pocket expenses and travel costs. This structural disparity means that for rural families, a health shock is more likely to precipitate a medical poverty trap. In this scenario, high out-of-pocket costs not only trigger immediate poverty relapse but also sever the continuous resource supply essential for education, creating a vicious cycle that compromises intergenerational mobility [12].

From an international comparative perspective, the impact of health spending on poverty vulnerability is shaped by the social welfare architecture. Similar to evidence from Bangladesh and Nigeria, where reliance on direct out-of-pocket payments pushes households into poverty [38,39], rural China faces similar challenges. However, China’s rapidly aging population adds a unique layer of complexity, intensifying the burden of chronic disease management on household finances. The result is that health spending functions as a critical negative moderator: it erodes the financial foundation necessary for educational investments to yield returns. When health risks are not mitigated by adequate social protection, the potential of education to prevent relapse into poverty is significantly neutralized. Based on the preceding analysis, we propose the following hypothesis.

H6: Household health expenditure attenuates the mitigating effect of educational human capital on the risk of poverty relapse.

**Fig 1:**
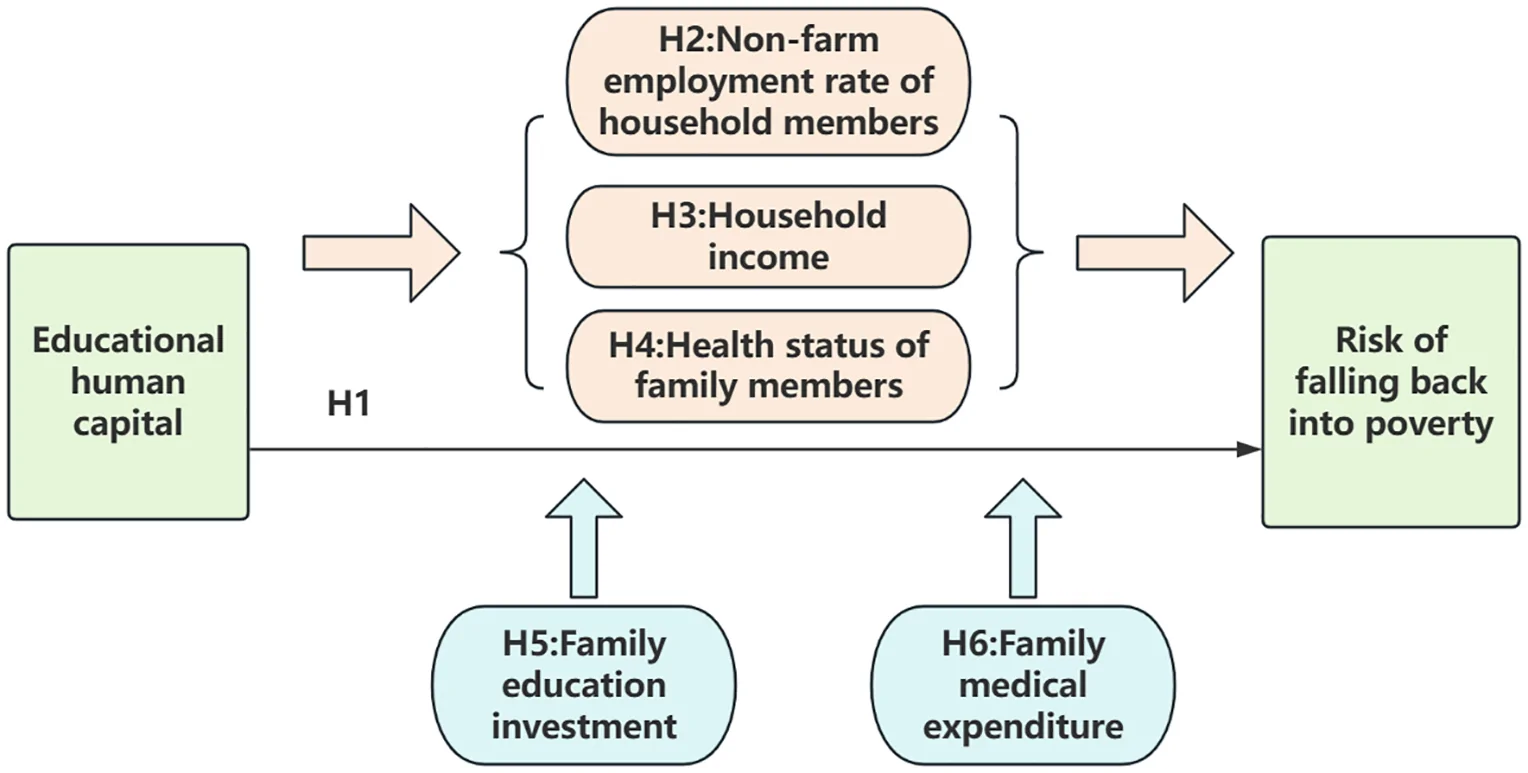
The theoretical analysis framework of this paper.

Fig 1 shows the overall research framework of this paper.

## 4. Research design

### 4.1 Model setting

#### 4.1.1 Two-way fixed effect regression model.

Given the substantial heterogeneity in access and utilization of educational resources across households and the associated divergence in educational outcomes, this study acknowledges the potentially confounding impact of time-varying macro factors, including policy reforms, economic fluctuations, and socio-contextual shifts, on the dynamics of poverty relapse. To address these complexities, a two-way fixed effects framework is employed to disentangle the causal mechanisms linking educational human capital to poverty vulnerability. Specifically, individual-specific fixed effects are integrated to neutralize the impact of time-invariant household attributes (e.g., geographic location, parental baseline characteristics) on dependent variables, thereby isolating within-family variations in educational outcomes. Concurrently, temporal fixed effects are incorporated to capture aggregate shocks and policy interventions that evolve across observation periods, ensuring robust identification of time-varying educational impacts. This methodological approach enhances analytical precision by concurrently controlling for both household-level heterogeneity and macro-level exogenous fluctuations, thereby providing a more nuanced estimation of how educational investments mediate poverty recurrence risks under dynamic environmental conditions. The following bi-directional fixed effect model is constructed:


$$\begin{array}{c} {PASI}_{it}=\alpha_{\text{0}}+\alpha_{\text{1}}{educ}_{it}+\sum_{i=\text{1}}^{k}\theta_{i}{ctrol}_{it}+\mu_{i}+\delta_{t}+\varepsilon_{it} \end{array}$$ (1)

In this model specification, ${\text{PASI}}_{\text{it }}$ denotes the poverty relapse risk index for household i during year t, while ${\text{educ}}_{\text{it}}$ quantifies the educational human capital stock of the same household in the corresponding period. ${\text{ctrol}}_{\text{it}}$ is a series of control variables for the i family in the t year; $\alpha_{\text{1}}$ is the parameter to be estimated; $\alpha_{\text{1}}$ is a constant term; $\epsilon_{\text{it}}$ is a random disturbance term. $\mu_{\text{i}}$ represents individual effect; $\delta_{\text{t}}$ represents the time effect.

#### 4.1.2 Mediation effect model.

This paper studies the realization path of educational human capital to reduce the risk of relapse into poverty from three aspects: non-agricultural employment rate, family income and health status of family members. Stepwise regression method was used to construct the following mechanism model:


$$\begin{array}{c} M_{it}=\beta_{\text{0}}+\beta_{\text{1}}{educ}_{it}+\sum_{i=\text{1}}^{k}\theta_{i}{ctrol}_{it}+\mu_{i}+\delta_{t}+\varepsilon_{it} \end{array}$$ (2)

$$\begin{array}{c} {PASI}_{it}=\gamma_{\text{0}}+\gamma_{\text{1}}{educ}_{it}+\gamma_{\text{2}}M_{it}+\sum_{i=\text{1}}^{k}\theta_{i}{ctrol}_{it}+\mu_{i}+\delta_{t}+\varepsilon_{it} \end{array}$$ (3)

Where, M is the mechanism variable, including non-farm employment rate of family members (empl), family income status (inco) and health status of family members (heal). When both coefficients $\beta_{\text{1}}$ and values $\gamma_{\text{2}}$ were significant, the mediation effect was significant, and then Bootstrap test was used to test the mediation effect. The interpretation of other variables is consistent with equation (1).

#### 4.1.3 Moderation effect model.

The moderating effect test is a key analysis method to explore the influence of educational human capital on the risk of relapse into poverty. With reference to the practice of Liu (2024), the following moderation effect model is constructed [8]:


$$\begin{array}{c} {Rrp}_{it}=\lambda_{\text{0}}+\lambda_{\text{1}}{educ}_{it}+\lambda_{\text{2}}W_{it}+\lambda_{\text{3}}educ\times W+\sum_{i=\text{1}}^{k}\theta_{i}{ctrol}_{it}+\mu_{i}+\delta_{t}+\varepsilon_{it} \end{array}$$ (4)

Where, W is the regulating variable, including family education investment (eexp) and family medical expenditure (mexp). educ×W represents the multiplicative interaction between the primary explanatory variable and the moderating variable W. When coefficients 1 and 3 are both significant, it indicates that the relationship between educational human capital and the risk of relapse into poverty is regulated by the moderating variables. On this basis, if 1 and 3 are of the same sign, it indicates that the moderating variable strengthens the influence of educational human capital on the risk of relapse into poverty, and vice versa, it indicates that the moderating variable weakens the influence.

### 4.2 Description of variables

#### 4.2.1 Explained variables.

The dependent variable in this study is the risk index for relapse into poverty (Rrp).Table 1 summarizes the multidimensional framework used to construct the composite risk index for relapse into poverty. Conceptually, the risk of relapse into poverty refers to the latent likelihood that a household will fall into or return to poverty in the future. Since household survey data does not directly capture this likelihood, this study uses a multidimensional composite index as an empirical proxy, where higher Rrp values indicate a greater relative likelihood of future poverty relapse. The poverty vulnerability literature supports this measurement strategy, generally treating future poverty risk as a forward-looking latent construction and evaluating it through observable household conditions when direct probability estimation proves infeasible. Researchers often conceptualize vulnerability as the likelihood of falling into poverty in the future, typically inferring household risk and vulnerability from observed socioeconomic characteristics rather than directly measuring probability [40–42]. This approach also aligns with the sustainable livelihoods perspective, which highlights the role of household assets and capabilities in coping with shocks [43], and with multidimensional poverty research, which demonstrates that income alone cannot adequately capture welfare deprivation [10]. Moreover, empirical studies widely employ composite indicators to measure latent vulnerability and resilience [44–46].

**Table 1:** The indicator system for the risk of relapse into poverty.

Dimension	Obstacle factor	Indicator Name	Indicator calculation and threshold	Indicator direction
Material risk	C_1_	Per capita income of rural/urban households	Total family income/family size	negative
	C_2_	Total cash and deposits	Survey direct acquisition	negative
	C_3_	Net household property	Survey direct acquisition	negative
	C_4_	Value of consumer durables	Survey direct acquisition	negative
Financial risk	C_5_	Non-mortgage financial liabilities	positive	positive
	C_6_	Own assets in financial products	Survey direct acquisition	negative
Social risk	C_7_	Participation rate of cooperative medical Care (%)	Number of health insurance participants/Number of households	negative
	C_8_	Participation rate of Social Endowment Insurance (%)	Number of pension insurance participants/Number of families	negative
Human resource risk	C_9_	Household share of migrant workers (%)	Number of labor force/Number of family members	negative
	C_10_	Proportion of unhealthy family members (%)	Number of unhealthy family members/total number of family members	positive
	C_11_	Level of education per adult	Illiteracy/semi-illiteracy = 0, primary school = 6, junior high school = 9, high school/technical school/vocational high school = 12, junior college = 15, bachelor’s degree = 16, master’s degree = 19, doctor’s degree = 22	negative
Life risk	C_12_	Whether to allocate collective land	Yes = 1, no = 0	negative
	C_13_	Cooking water	Tap water = 1, bottled water/purified water/filtered water = 2, other = 0	negative
	C_14_	Per capita living space	Housing area/Total family size	negative
	C_15_	Medical expenditure	Survey direct acquisition	positive

Grounded in the Sustainable Livelihoods Approach [47] and the Capability-Vulnerability Framework [48], we construct Rrp from five primary dimensions: material risk, financial risk, social risk, human resource risk, and life risk. These dimensions are operationalized in CFPS through 15 secondary metrics chosen based on their theoretical relevance and data availability. The entropy method is then applied to determine the objective weights, which are aggregated into the final composite metric. In this study, the entropy method is used as a weighting and aggregation tool rather than a direct estimator of the probability.

(1) Material risk. Material risk constitutes the primary economic buffer against exogenous shocks. This dimension is captured by four negative indicators: per capita household income (C1), total cash and deposits (C2), net household property (C3), and the value of consumer durables (C4). Income and liquid assets (C1, C2) represent immediate consumption possibilities and the capacity to smooth consumption during emergencies [49]. Meanwhile, net property and durables (C3, C4) serve as proxies for permanent income and long-term asset resilience, determining the household’s ability to withstand sustained economic stress [50].
(2) Financial risk. The financial dimension assesses the structural balance between risky liabilities and protective assets. We select non-mortgage financial liabilities (C5) as a positive indicator, as high unsecured debt exacerbates liquidity constraints and increases the probability of shock-induced poverty traps [51]. Conversely, financial asset holdings (C6) function as a negative indicator, reflecting the household’s capacity for risk diversification and inter-temporal resource allocation, which are crucial for mitigating financial distress [52].
(3) Social risk. Social integration reflects the extent to which formal protection systems buffer against lifelong risks. This dimension is measured by the coverage rates of basic medical insurance (C7) and basic pension insurance (C8). Medical insurance reduces the risk of impoverishment due to catastrophic health expenditures [53], while pension coverage provides essential old-age income security and enhances intergenerational risk-sharing. Both are treated as negative indicators, where higher participation signals greater institutional protection against relapse into poverty.
(4) Human resource risk. Human capital determines the earning potential and adaptability of a household. This dimension includes three indicators: the household share of migrant workers (C9), the proportion of unhealthy members (C10), and the average years of adult schooling (C11). Migrant labor participation (C9) and educational attainment (C11) are negative indicators, representing income diversification and labor market competitiveness. In contrast, the proportion of unhealthy members (C10) is a positive indicator, as poor health erodes labor productivity and precipitates medical financial burdens [54].
(5) Life risk. Life risk captures physical quality of life and access to essential productive and subsistence resources. This dimension includes the allocation of collective land (C12) alongside indicators of basic housing and utility conditions (C13-C15). Collective land functions as a critical livelihood safety net, particularly in rural contexts, providing both productive inputs and social security; Hence, it is encoded as a negative indicator. Similarly, access to secure living amenities is integral to long-term well-being [14], with deprivation in this domain signaling heightened vulnerability to poverty relapse.

Given the heterogeneous units and orientations of these metrics, we normalize each variable using min-max scaling:


$$\begin{array}{c} \begin{array}{c} \text{ positive indicators}:\text{Yij}=\frac{\text{Xij}-\text{Xjmin}}{\text{Xj}\text{max}-\text{Xjmin}} \end{array} \end{array}$$ (5)

$$\begin{array}{c} \begin{array}{c} \text{negative indicators}:\text{Yij}=\frac{\text{Xj}\text{max}-\text{Xij}}{\text{Xi}\text{max}-\text{Ximin}} \end{array} \end{array}$$ (6)

Where, n is the number of dimensions; m is the number of indicators in the corresponding dimension. Yij is the standardized value of the JTH evaluation index of the i family; Xij is the original value of the JTH evaluation index of the i family; Xjmax and Xjmin are the maximum and minimum values of the JTH evaluation index respectively.

The entropy value Hj of the JTH index is calculated from equation (7), and the entropy weight wj of the JTH index is calculated from equation (8).


$$\begin{array}{c} \text{Hj}=-\text{k}\sum_{\text{i}=\text{1}}^{\text{n}}\text{fij}\text{ln}\text{f}\text{ij},(\text{i}=\text{1},\text{2},\ldots ,\text{n};\text{j}=\text{1},\text{2},\ldots ,\text{m}) \end{array}$$ (7)

$$\begin{array}{c} \begin{array}{c} \text{wj}=\frac{\text{1}-\text{Hj}}{\text{m}-\sum_{\text{j}=\text{1}}^{\text{m}}\text{Hj}},(\text{j}=\text{1},\text{2},\ldots ,\text{m}) \end{array} \end{array}$$ (8)

Where, $\text{k}=\frac{\text{1}}{\text{ln}\text{n}}$, $\text{fij}=\frac{\text{yij}}{\sum_{\text{i}=\text{1}}^{\text{m}}\text{yij}}$. fij is the proportion of the index value of the i item of the j index.

After calculating the weight of each index, the five dimensions are weighted and summed by the comprehensive weight of the index to calculate the risk index of each household relapse into poverty. The calculation formula is as follows:


$$\begin{array}{c} \begin{array}{c} \text{Rrp}=\sum_{\text{i}=\text{1}}^{\text{n}}\sum_{\text{j}=\text{1}}^{\text{m}}\text{yijwij} \end{array} \end{array}$$ (9)

Where, Rrp is the risk index of households relapse into poverty; wij is the weight value of the ith-dimension JTH index.

#### 4.2.2 Explanatory variables.

The explanatory variable of this paper is the level of educational human capital, which is measured by the per capita education years of adults based on the practice of Li [12]. For the eight categories of “illiterate/semi-illiterate, primary school, junior high school, high school (junior/technical/vocational high school), junior college, bachelor’s degree, master’s degree, doctor’s degree”, the corresponding years of education are 0, 6, 9, 12, 15, 16, 19 and 22 years respectively.

#### 4.2.3 Mediating variables.

(1) Non-Farm Employment Rate (empl). This study employs the share of adult household members engaged in non-agricultural employment as the proxy for assessing household labor market diversification. (2) Household Income (inco). Per capita household income serves as the primary indicator for quantifying economic welfare levels. (3) Health Status (heal). Household members’ health conditions are evaluated using a five-tiered self-assessment scale, where numerical values are assigned as follows: “excellent” = 5, “very good” = 4, “moderately healthy” = 3, “average” = 2, and “poor” = 1. This ordinal metric captures dimensional variations in physical well-being across family units.

#### 4.2.4 Moderating variables.

(1) Family Education Investment (eexp). this paper selects the family’s education and training expenditure in the past 12 months as a measure.
(2) Family Medical Expenditure (mexp). this paper selects the family’s medical expenditure in the past 12 months as a measure.

#### 4.2.5 Control variables.

Table 2 delineates variable definitions and descriptive statistics. To account for potential confounding influences of household heterogeneity and regional disparities, this study incorporates the following control variables: (1) Family Dependency Ratio (fraise). Quantified as the proportion of household members aged <14 or >65 years, this metric captures demographic dependency burdens [55] (2) Household Head Age. Age of the primary income earner is included, as advanced age may attenuate labor market competitiveness and income-generating capacity, thereby affecting antipoverty efficacy [56]. (3) Regional Economic Development (lnGDP). Provincial per capita GDP (log-transformed) serves as the proxy for macroeconomic context, given its documented correlation with resident income levels and poverty dynamics [6]. (4) Land Endowment. A binary indicator (1 = presence of collective agricultural land; 0 = otherwise) reflects the insurance value of rural landholdings against income volatility, with arable land hypothesized to stabilize household income during economic shocks [57]. (5) Household Head Gender (gen). Gender is dichotomized (1 = male; 0 = female), reflecting empirical evidence suggesting male-headed households exhibit stronger risk resilience and poverty avoidance capabilities [58]. These covariates collectively mitigate omitted variable bias by controlling for demographic, economic, and institutional factors that may confound the relationship between educational human capital and poverty relapse risks.

**Table 2:** Variable definitions and descriptive statistics.

Variable type	Variable Name	Symbol	Mean	Std.Dev.	Min	Max
Explained variable	Risk of falling back into poverty (index)	Rrp	0.142	0.095	0.011	0.605
Explanatory variable	Educational human capital(years)	educ	7.668	4.733	0	19
Mediating variables	The proportion of family members participating in employment(proportion)	empl	0.163	0.251	0	1
	Per capita household income (10,000 yuan)	inco	9.510	1.137	0	14.230
	Health status of family members (1–5)	heal	2.807	1.202	1	5
Moderating variables	Family education investment (Log)	lneexp	3.845	4.265	0	12.206
	Share of household medical expenditure (proportion)	mexp	0.191	0.278	0	1
Control variable	Family dependency ratio (proportion)	fraise	0.145	0.325	0	1
	Land endowment (dummy)	land	0.629	0.483	0	1
	Age of head of household (years)	age	52.860	13.150	17	90
	The number of the economic development level of the region (Log)	lnGDP	10.870	0.401	10.180	12.010
	Gender of head of household (male = 1, female = 0)	gen	0.552	0.497	0	1

Variables prefixed with “ln” are log-transformed.

#### 4.2.6 Correlation and multicollinearity diagnostics.

Table 3 presents the matrix of Pearson correlation coefficients among the variables. Pairwise correlations indicate that the risk index of relapse into poverty (Rrp) is negatively correlated with educational human capital (educ), per capita income (inco), non-farm employment share (empl), family education investment (lneexp), land endowment (land), and regional economic development (lnGDP), while positively associated with worse health status (heal), higher medical expenditure share (mexp), and a higher dependency ratio (fraise). Importantly, the absolute correlations among regressors are generally below 0.5, with the highest value around 0.47 (between educ and inco), well below the conventional threshold (0.8) that would signal serious multicollinearity concerns. This suggests that explanatory variables, including educational human capital, are not simple linear combinations of each other and contain sufficient discriminative information for regression analysis.

**Table 3:** Matrix of Pearson correlation coefficients.

Variables	Rrp	educ	empl	inco	heal	lneexp	mexp	fraise	land	age	lnGDP	gen
Rrp	1.000	−0.280	−0.220	−0.410	0.270	−0.190	0.290	0.240	−0.180	0.090	−0.260	−0.070
educ	−0.280	1.000	0.310	0.470	−0.190	0.360	−0.100	−0.210	−0.090	0.180	0.330	0.060
empl	−0.220	0.310	1.000	0.390	−0.140	0.170	0.040	−0.120	−0.050	−0.110	0.150	0.080
inco	−0.410	0.470	0.390	1.000	−0.220	0.290	0.120	−0.250	−0.070	0.050	0.410	0.030
heal	0.270	−0.190	−0.140	−0.220	1.000	−0.080	0.210	0.180	−0.030	0.160	−0.090	−0.020
lneexp	−0.190	0.360	0.170	0.290	−0.080	1.000	0.110	−0.100	−0.020	0.070	0.180	0.010
mexp	0.290	−0.100	0.040	0.120	0.210	0.110	1.000	0.070	−0.010	0.040	0.030	−0.010
fraise	0.240	−0.210	−0.120	−0.250	0.180	−0.100	0.070	1.000	0.050	0.220	−0.130	−0.030
land	−0.180	−0.090	−0.050	−0.070	−0.030	−0.020	−0.010	0.050	1.000	0.030	−0.080	0.020
age	0.090	0.180	−0.110	0.050	0.160	0.070	0.040	0.220	0.030	1.000	−0.040	0.190
lnGDP	−0.260	0.330	0.150	0.410	−0.090	0.180	0.030	−0.130	−0.080	−0.040	1.000	0.020
gen	−0.070	0.060	0.080	0.030	−0.020	0.010	−0.010	−0.030	0.020	0.190	0.020	1.000

Table 4 presents the variance inflation factors (VIF) for the main variables. The VIF statistics further confirm that multicollinearity is not a serious problem in our empirical specifications. All variables exhibit VIF values well below the commonly used threshold of 10, with an average VIF of only 1.63. Educational human capital (educ) shows a VIF of 2.21, and per capita income (inco) has a VIF of 2.45, indicating moderate but acceptable correlation with other regressors. These results imply that the accuracy of coefficient estimates is unlikely to be substantially distorted by multicollinearity, and that the inclusion of multiple mechanisms and control variables is statistically justified.

**Table 4:** The variance inflation factors (VIF) for the main variables.

Variables	inco	educ	lnGDP	empl	fraise	age	lneexp	heal	mexp	land	gen
VIF	2.45	2.21	2.03	1.78	1.61	1.47	1.43	1.35	1.29	1.18	1.12
1/VIF	0.408	0.453	0.493	0.562	0.622	0.680	0.699	0.742	0.774	0.847	0.893

### 4.3 Data sources

This study utilizes longitudinal data from the 2014, 2016, 2018, and 2020 waves of the CFPS, administered by Peking University’s Institute of Social Science Survey. The CFPS conducts multilevel data collection at individual, household, and community levels to document sociodemographic, economic, educational, and health transitions across China. Covering 25 provincial-level administrative regions representing approximately 95% of the national population, the survey provides nationally representative longitudinal observations. Household-level data integration followed a rigorous protocol: (1) Merging household economic and adult respondent databases using unique household identifiers (fid); (2) Restricting analyses to households participating in all four survey waves (2014–2020); (3) Screening core variables including educational attainment, income, and expenditure metrics. Missing values were imputed through linear interpolation to maintain a balanced panel structure, ensuring parameter estimation consistency. The final analytical sample comprises 2,990 households (11,960 individual observations) with complete six-year follow-up records. To mitigate macroeconomic confounding, provincial GDP per capita statistics sourced from the China Statistical Yearbook were spatially and temporally matched to CFPS observations, enabling control for regional economic development impacts on poverty vulnerability. This methodological framework facilitates rigorous examination of educational human capital’s poverty-alleviating mechanisms while accounting for both micro-level household dynamics and macro-level contextual influences.

## 5. Analysis of empirical results

### 5.1 Baseline regression analysis

Table 5 presents the baseline regression results. These findings provide robust evidence that educational human capital mitigates the risk of falling back into poverty. This effect holds across diverse model specifications. In the unconditional model (Column 1), the coefficient for educational attainment (educ) is −0.132. This estimate is statistically significant at the 1% level. Including household and regional covariates (Column 2) attenuates the coefficient to −0.063. However, it remains highly significant. This confirms that the protective effect of educational human capital persists even after accounting for observable confounding. Crucially, column 4 employs a fixed-effects specification. This model rigorously controls for unobserved individual and temporal heterogeneity. Here, the coefficient stabilizes at −0.046. It also maintains a significance of 1% level. This consistent negative relation highlights the robustness of our central finding. Educational human capital acts as a crucial stabilizer. It significantly reduces the probability of relapse to poverty regardless of the model specification.

**Table 5:** Baseline regression results.

Variables	(1)	(2)	(3)	(4)
educ	−0.132***	−0.063***	−0.059***	−0.046***
	(0.009)	(0.007)	(0.031)	(0.026)
fraise		0.086***		0.043***
		(0.008)		(0.010)
age		0.133***		0.001
		(0.008)		(0.010)
lnGDP		0.005		0.002
		(0.006)		(0.024)
land		−0.733***		−0.700***
		(0.007)		(0.011)
gen		−0.065***		0.468
		(0.013)		(0.286)
Constant	−0.000**	0.036***	−0.064***	−0.236**
	(0.009)	(0.009)	(0.012)	(0.168)
Time effect	No	No	Yes	Yes
Individual effect	No	No	Yes	Yes
Observations	11,960	11,960	11,960	11,960
R^2^	0.018	0.570	0.017	0.320
F-statistic			38.243	458.798

Asterisks *, **, and *** denote statistical significance at the 10%, 5%, and 1% levels (p-values), respectively. Robust standard errors, clustered at the household level, are presented in parentheses below the coefficient estimates as referenced in subsequent tables.

Controlling for control variables, the results highlight several significant determinants of vulnerability. First, the household dependency ratio emerges as a critical risk factor. A higher burden of dependent members strains household disposable income. This pressure increases susceptibility to poverty relapse [59]. Second, gender dynamics play a significant role. Households with female heads exhibit higher vulnerability. This likely stems from structural inequalities in resource access and labor market opportunities [60,61]. In turn, economic context and asset endowments exert protective effects. Regional economic development (proxied by GDP) is negatively associated with relapse risk. This suggests that broader economic growth has enhanced household financial resilience. Similarly, land endowment functions as a “natural insurance” mechanism. It provides essential income security. This security effectively mitigates vulnerability during economic downturns.

### 5.2 Discussion of endogeneity

Although the baseline two-way fixed-effects model controls for unobserved individual heterogeneity and time effects, the estimated relationship between educational human capital and the risk of falling back into poverty may still suffer from endogeneity. Such endogeneity may arise from reverse causality, omitted variables, or dynamic persistence in poverty relapse risk. To further verify the reliability of the benchmark results, this paper conducts two additional analyses: a one-period lagged explanatory variable approach and a dynamic panel System GMM estimation.

#### 5.2.1 One-period lag of the explanatory variable.

To address potential endogenous bias arising from the two-way causality between educational human capital and poverty relapse risk, which could introduce biased coefficient estimates and compromise model validity, this study employs a lagged instrumental variable approach. Recognizing that the lagged independent variable is less likely to be contemporaneously influenced by the dependent variable, the analysis utilizes a one-period lagged measure of human capital as the instrumental variable to mitigate endogeneity concerns. Robustness verification through a one-period delayed proxy variable for human capital development is conducted, with results presented in column (1) of Table 6. The instrumental variable demonstrates sufficient strength as evidenced by an F-statistic exceeding 10, passing the weak instrument test and confirming its adequacy. The statistically significant negative coefficient at the 1% confidence level for educational human capital indicates effective control of reciprocal causation issues, thereby reinforcing the study’s conclusion regarding the protective effect of education against poverty recurrence. This methodological approach enhances estimation accuracy while substantiating the causal interpretation that educational attainment reduces vulnerability to re-entering poverty.

**Table 6:** Endogeneity test results.

Variables	(1)	(2)
	One-period lag of educ	system GMM
L.Rrp		0.398***
		(0.037)
educ	−0.069***	−0.021***
	(0.008)	(0.006)
Constant	0.054***	0.511***
	(0.011)	(0.087)
Observations	8,970	7800
R^2^	0.553	
F-statistic		
Wald χ² (overall)		341.2***
AR(1) test (z, p-value)		−3.18 (0.001)
AR(2) test (z, p-value)		0.79 (0.430)
Hansen J (χ²(55), p-value)		52.4 (0.580)

#### 5.2.2 Dynamic panel System GMM estimation.

To further address potential endogeneity due to omitted variables, reverse causality, and dynamic persistence of poverty relapse risk, the dynamic panel system GMM method is used for additional estimation. The model is specified as follows:


$$\begin{array}{c} Rrp_{it}=\alpha Rrp_{i,t-\text{1}}+\beta educ_{it}+\gamma X_{it}+\mu_{i}+\lambda_{t}+\epsilon_{it} \end{array}$$ (10)

In this specification, both the lagged dependent variable $Rrp_{i,t-\text{1}}$ and educational human capital $educ_{it}$ are treated as potentially endogenous variables, and they are instrumented by their second and higher order lags. The remaining control variables are considered either weakly exogenous or strictly exogenous. For ease of comparison with our baseline results, the System GMM estimation results and corresponding diagnostic tests are presented together in Table 6.

Column (2) of Table 6 presents the results from the dynamic system GMM specification. After controlling for the lagged risk index and a comprehensive set of household and regional covariates, the coefficient for educ remains negative and statistically significant at the 1% level. The estimated coefficient of −0.021 implies, ceteris paribus, that each additional year of average adult schooling is associated with a statistically and economically significant reduction in the probability of falling back into poverty. This finding is closely aligned with the baseline regression results, suggesting that the risk-mitigating effects of education are robust even after accounting for potential endogeneity.

### 5.3 Robustness test

#### 5.3.1 Tail reduction.

To mitigate the confounding influence of outliers on empirical estimates, this study implemented 1% winsorization for all continuous variables. Column (1) in Table 7 reports the regression outcomes following this data preprocessing step. Even after truncating extreme values at both tails of the distribution, the estimated coefficient for educational human capital retains its statistically significant negative association with poverty relapse risk at the 1% threshold. This finding underscores the robustness of our central conclusion across alternative data specifications, as the protective effect of educational endowments persists despite reduced sensitivity to outlier observations. The consistency between unadjusted and winsorized results validates the analytical framework by demonstrating that the observed poverty-reduction mechanism is not an artifact of extreme value distortions, thereby reinforcing the reliability of causal inferences regarding educational human capital’s role in poverty alleviation.

**Table 7:** Robustness test results.

Variables	(1) 1% winsorization	(2) Equal-weighted Rrp	(3) High-dimensional fixed effects
educ	−0.043***	−0.042***	−0.036**
	(0.007)	(0.026)	(0.015)
Constant	0.401***	−0.361**	0.518***
	(0.076)	(0.159)	(0.126)
Time effect	Yes	Yes	Yes
Individual effect	Yes	Yes	Yes
Province effect	No	No	Yes
Province × year effect	No	No	Yes
Observations	11,960	11,960	11,960
R^2^	0.317	0.301	0.336

#### 5.3.2 Alternative construction of the dependent variable.

The benchmark regression constructs the poverty relapse risk index using the entropy weighting method. To test whether the empirical results are sensitive to the weighting scheme, this paper reconstructs the dependent variables by assigning equal weights to each indicator and then re-estimating the model. Column (2) of Table 7 presents the corresponding results. The coefficient for educational human capital remains significantly negative at the 1% level, indicating that the core conclusions do not depend on the specific weighting method used to construct the poverty relapse risk index.

#### 5.3.3 High-dimensional fixed effects.

Considering that education levels and poverty relapse risk may vary systematically across regions and over time, the benchmark specification may still be affected by more nuanced spatial heterogeneity. To further control for such unobservable factors, in this paper we introduce higher dimensional fixed effects and re-estimate the model. Specifically, province fixed effects and province-by-year interaction fixed effects are added on the basis of the benchmark specification. The estimation result is shown in column (3) of Table 7. The coefficient of educational human capital remains significantly negative, suggesting that the poverty-reducing effect of educational human capital is robust even under a more stringent fixed-effects structure.

In summary, the results of the above three robustness tests are in high agreement with the benchmark regression, which further confirms that the conclusion that educational human capital significantly reduces the risk of falling back into poverty is robust and reliable.

### 5.4 Mediation effect test

Table 8 presents the mediation effect analysis outcomes. Columns (1)-(2) display the relationship between educational human capital and household members’ non-farm employment rate, along with the subsequent impact of employment status on poverty recurrence risk.The regression results indicate that educational human capital exhibits a significant negative association with the non-farm employment rate (β = −0.031, p < 0.01), while a higher employment rate significantly reduces the risk of relapse into poverty (β = −0.828, p < 0.01). The product of these two negative coefficients yields a positive indirect effect. Through Bootstrap test, 99% confidence intervals do not contain 0, indicating that the indirect effect is significant and the existence of mediation effect is verified. The mediation effect of non-farm employment rate of household members is 0.026, accounting for 55.80% of the total effect. Therefore, the non-agricultural employment rate of family members, as a mechanism, can effectively link the inhibitory effect of educational human capital on the risk level of relapse into poverty. Relevant studies have shown that educating human capital improves family members’ chances of entering the non-farm job market. Non-farm employment generally pays more and is more stable than agricultural employment, thus increasing the economic resilience of households and reducing the risk of households relapse into poverty, indicating the key mechanism role of non-farm employment in the relationship between education and the risk of relapse into poverty [62]. At the same time, education helps family members to obtain non-agricultural jobs, which improves their income level and anti-risk ability. Especially in the case of unstable agricultural income, non-agricultural income becomes the main support for poverty alleviation [57]. As a result, non-farm employment provides rural households with a more reliable source of income than agricultural income, effectively linking education to long-term poverty alleviation.

**Table 8:** Results of the mediation effect test.

Variables	(1)	(2)	(3)	(4)	(5)	(6)
	empl	RRP	inco	RRP	heal	RRP
educ	−0.031***		−0.040***		−0.012***	
	(0.005)		(0.004)		(0.003)	
empl		−0.828***				
		(0.011)				
inco				−0.076***		
				(0.008)		
heal						−0.261***
						(0.007)
Control variables	Control	Control	Control	Control	Control	Control
Time effect	Yes	Yes	Yes	Yes	Yes	Yes
Individual effect	Yes	Yes	Yes	Yes	Yes	Yes
Constant	0.444***	0.404***	8.748***	0.429**	−0.005	−0.237
	(0.006)	(0.009)	(0.232)	(0.180)	(0.224)	(0.158)
Observations	11,960	11,960	11,960	11,960	11,960	11,960
R^2^	0.127	0.710	0.131	0.323	0.005	0.398
Bootstrap	[0.018,0.033]		[0.015,0.024]		[0.012,0.024]	
	(z = 6.49,p = 0.000)		(z = −8.60,p = 0.000)		(z = −5.69,p = 0.000)	

Columns (3) and (4) of Table 8 present the mediation pathway analysis for household income. The results show a significant negative coefficient for education on income (β = −0.040, p < 0.01) and a significant negative effect of income on poverty relapse risk (β = −0.076, p < 0.01). Bootstrap testing confirms significant indirect effects as all 99% confidence intervals exclude zero ([0.015, 0.024]), validating the mediation mechanism through economic status. The income-mediated pathway accounts for 6.67% of total effects with an effect size of 0.003, indicating education contributes to poverty reduction and economic stabilization via enhanced individual/family earning capacity. This mechanism aligns with established findings: education strengthens labor market competitiveness and income potential, generating sustained household income growth. Elevated income levels enable reinforcing investments in human capital and health infrastructure, thereby solidifying economic resilience and stabilizing poverty reduction outcomes [63]. Cross-sectional evidence reveals children from higher-income households exhibit stronger labor market performance compared to peers from low-income backgrounds, creating generational income advancement effects. Concurrent income growth enhances poverty alleviation sustainability as educational attainment improves individual earning power, with accumulated resources facilitating expanded household investments that stabilize economic foundations and mitigate poverty relapse risks [2]. For rural populations, education serves as a critical poverty escape mechanism by directly elevating family income [64]. These findings collectively demonstrate household income functions as a pivotal transmission channel through which educational human capital reduces vulnerability to poverty recurrence.

Columns (5) and (6) of Table 8 present the mediation analysis concerning health status. Educational attainment shows a significant negative association with health indicators (β = −0.012, p < 0.01), while health status significantly reduces poverty relapse risk (β = −0.261, p < 0.01). Bootstrap results with 99% confidence intervals excluding zero ([0.012, 0.024]) confirm significant indirect effects, validating health as a transmission mechanism. The health-mediated pathway accounts for 6.70% of total effects with an effect size of 0.003, indicating education influences household economic welfare not only through direct income channels but also via health improvements. Extant literature supports this pathway: educational attainment enhances health literacy and medical decision-making capabilities, thereby elevating overall household health levels. Health functions as a critical poverty prevention mechanism by reducing illness-related income shocks and solidifying poverty escape outcomes [65]. Rural contexts demonstrate particularly strong effects, with education correlating with improved self-assessed health metrics and reduced depression risks [31]. Intergenerational dynamics further reinforce this mechanism, as parental education and health status directly shape children’s human capital development, while child health outcomes influence parental labor supply and household economic conditions [30]. Optimal health status enhances work capacity and earning potential, enabling households to maintain economic stability and better withstand financial disruptions [66]. Consequently, household health indicators serve as a vital transmission channel through which education strengthens economic productivity and resilience against poverty recurrence, thereby stabilizing anti-poverty achievements.

### 5.5 Moderation effect test

Table 9 presents the moderation effect analysis results. As shown in column (2), the regression coefficient for household educational expenditure (eexp) is −0.015 (p < 0.01), indicating a significant negative relationship. The interaction term coefficient between educational attainment (educ) and eexp in column (3) reaches 0.012 (p < 0.01), suggesting that increased family educational investments significantly strengthen the poverty reduction effect of human capital development. Extant literature corroborates this mechanism: household educational expenditures enhance learning outcomes, vocational skill acquisition, and employment quality, all critical for sustaining poverty alleviation achievements [67]. Such investments often expand social networks and social capital accumulation, reinforcing households’ anti-poverty resilience. Educational investments improve descendants’ labor market competitiveness and social mobility prospects, thereby consolidating long-term poverty reduction gains [68]. Additional evidence indicates educational expenditures facilitate access to high-income occupations, elevate household income levels, and provide financial stability essential for maintaining poverty escape outcomes [8]. Collectively, these findings confirm that family educational investments positively moderate the protective effect of human capital against poverty relapse.

**Table 9:** Results of the moderation effect test.

Variables	(1)	(2)	(3)	(4)	(5)	(6)
	RRP	RRP	RRP	RRP	RRP	RRP
educ	−0.063***	−0.058***	−0.102***			
	(0.007)	(0.007)	(0.009)			
eexp		−0.015***	−0.017***			
		(0.002)	(0.002)			
educ×eexp			0.012***			
			(0.001)			
educ				−0.063***	−0.051***	−0.050***
				(0.007)	(0.007)	(0.007)
mexp					0.125***	0.116***
					(0.006)	(0.006)
educ×mexp						−0.035***
						(0.006)
Control variables	Control	Control	Control	Control	Control	Control
Time effect	Yes	Yes	Yes	Yes	Yes	Yes
Individual effect	Yes	Yes	Yes	Yes	Yes	Yes
Constant	0.036***	0.097***	0.102***	0.036***	0.028***	0.027***
	(0.009)	(0.011)	(0.011)	(0.009)	(0.009)	(0.009)
Observations	11960	11960	11960	11960	11960	11960
R^2^	0.564	0.568	0.570	0.564	0.578	0.580
F-statistic	2576.112	2240.781	1980.718	2576.112	2341.851	2059.209

As shown in column (5), the regression coefficient for household medical expenditure (mexp) is 0.125 (p < 0.01). The interaction term coefficient between educ and mexp in column (6) reaches −0.035 (p < 0.01), implying that elevated medical expenditures significantly undermine the poverty prevention effect of educational human capital. Research attributes this to the economic burden imposed by high healthcare costs, which counteracts the stabilizing influence of human capital on household finances [69]. This burden is particularly severe for low-income households, where medical expenses deplete limited resources and neutralize the economic advancement potential of educational investments. Such households face heightened vulnerability to poverty recurrence due to medical expenditure shocks [7]. Moreover, excessive healthcare costs divert resources from productive investments, limit sustained educational development, and impair intergenerational poverty reduction capacity [70], thereby exacerbating long-term poverty transmission risks [71].

### 5.6 Heterogeneity analysis

#### 5.6.1 Quantile regression analysis.

Quantile regression analysis enables estimation of conditional parameter heterogeneity across the poverty relapse risk distribution, thereby revealing potential heterogeneities in educational human capital’s mitigating effects. By characterizing the full spectrum of dependent variable distributions, this methodological approach enhances empirical rigor through quantifying education-related poverty-alleviation mechanisms at varying risk thresholds. Table 10 displays the quantile regression analysis outcomes, with Columns (1) through (5) representing the 10th, 25th, 50th, 75th, and 90th percentiles of the poverty risk distribution spectrum. This specification allows examination of heterogeneous effects across different vulnerability thresholds. While quantile-specific coefficients exhibit notable deviations from the baseline OLS estimate, the educational human capital variable maintains consistent negative coefficients statistically significant at the 1% level across all quantiles. Notably, the marginal effect magnitude demonstrates an inverted U-shaped trajectory—declining from the 10th to the 50th percentile before increasing toward the 90th percentile. This non-linear pattern suggests two mechanisms: (1) At lower risk quantiles, initial educational investments primarily serve as preventive measures against poverty entry; (2) At higher risk quantiles, households having escaped absolute poverty may leverage improved economic conditions for enhanced educational investment, creating reinforcing cycles of human capital accumulation and income diversification [29]. Such dynamic marginal effects underscore the poverty-reduction strategy’s contextual dependency, wherein educational returns manifest differentially based on baseline risk levels and household endowment trajectories.

**Table 10:** Quantile regression results.

Variables	(1)	(2)	(3)	(4)	(5)
	q10	q25	q50	q75	q90
educ	−0.030***	−0.027***	−0.023***	−0.073***	−0.161***
	(0.002)	(0.003)	(0.005)	(0.010)	(0.021)
fraise	0.170***	0.155***	0.012***	0.131**	0.124***
	(0.026)	(0.008)	(0.005)	(0.051)	(0.022)
age	0.004	0.027***	0.073***	0.082***	0.271***
	(0.004)	(0.005)	(0.007)	(0.009)	(0.031)
lnGDP	−0.004**	−0.000	0.011**	0.023***	0.024
	(0.001)	(0.003)	(0.004)	(0.005)	(0.021)
fraise	−0.650***	−0.732***	−0.720***	−0.710***	−0.716***
	(0.003)	(0.012)	(0.006)	(0.011)	(0.022)
gen	−0.007**	−0.006	−0.006	−0.055***	−0.113***
	(0.003)	(0.004)	(0.009)	(0.015)	(0.031)
Constant	−0.638***	−0.515***	−0.058***	0.258***	0.896***
	(0.009)	(0.008)	(0.008)	(0.018)	(0.027)
Observations	11,960	11,960	11,960	11,960	11,960

The improvement of educational human capital is not without obstacles. For low-income families, the uncertainty of opportunity cost and future returns of education investment will affect education investment decisions [72]. In addition, the improvement of educational human capital also needs the support and assistance of the government, such as improving the educational human capital of poor families through public education expenditure, and improving the education and income status of beneficiary individuals in poor areas through the “two exemptions and one supplement” policy.

#### 5.6.2 Analysis of urban-rural heterogeneity.

Regression analysis of urban-rural heterogeneity in Table 11 reveals distinct impacts of educational human capital on poverty resurgence risks across settlement types. While educational attainment exhibits significant negative coefficients for both rural (β = −0.033, p < 0.01) and urban (β = −0.046, p < 0.01) households, the magnitude of urban effects underscores the greater poverty-mitigating potency of education in metropolitan contexts. This urban advantage stems from structural disparities in educational resource allocation, as urban schools typically benefit from superior infrastructure, qualified instructors, and curriculum offerings compared to their rural counterparts. Limited rural educational resources constrain human capital accumulation, thereby diminishing the economic returns to education for non-urban households [7]. Furthermore, the urban labor market’s premium on educational credentials amplifies the income-stabilizing effects of higher learning, with educated urban residents accessing higher-quality employment opportunities that enhance economic resilience [73]. Such systemic urban-rural divides in education quality and labor market integration create a virtuous cycle for urban households, where educational investments yield proportionally greater dividends in poverty prevention. These findings highlight the need for spatially targeted interventions that address rural educational deficits while leveraging urban education’s comparative advantages to optimize poverty reduction outcomes across settlement gradients.

**Table 11:** Results of urban-rural heterogeneity regression.

Variables	(1)	(2)
	urban	rural
educ	−0.046***	−0.033***
	(0.010)	(0.010)
Control variables	Control	Control
Time effect	Yes	Yes
Individual effect	Yes	Yes
Constant	0.018	0.038**
	(0.013)	(0.017)
Observations	5,967	5,858
R^2^	0.588	0.371

#### 5.6.3 Regional heterogeneity analysis.

Table 12 presents the findings of subgroup regression analyses examining regional disparities in the poverty-mitigating effects of educational human capital across eastern, central, and western China. Following geographical stratification of provincial samples, the study identifies pronounced regional heterogeneity in these relationships. Consistent with theoretical expectations, educational attainment demonstrates statistically significant negative coefficients in all regional subgroups, confirming its universal poverty-reduction function. Notably, the eastern region exhibits the strongest mitigating effect, followed by the western region, with the central region showing the weakest association.These divergences reflect underlying structural factors, including disparities in economic development, technological infrastructure, industrial specialization, and policy priorities across regions. The eastern region’s advantage stems from its higher-quality educational institutions, greater labor market integration, and stronger industry-education linkages, which amplify the economic returns to human capital. Conversely, the central region’s relatively weaker impact may relate to its intermediate level of development and less diversified economy. While the western region’s coefficient exceeds that of the central region, this finding aligns with targeted national development programs that have enhanced educational accessibility and rural revitalization efforts in western provinces. Geographical variations in coefficient magnitudes further highlight the moderating role of regional context: economic vitality and industrial sophistication in coastal areas enable more effective conversion of educational credentials into income-generating opportunities, thereby strengthening education’s poverty-preventative capacity. These results underscore the necessity of spatially differentiated anti-poverty strategies that account for regional disparities in human capital efficiency and developmental needs.

**Table 12:** Regression results for regional heterogeneity.

Variables	(1)	(2)	(3)
	Eastern	Central	Western
educ	−0.128***	−0.085*	−0.093**
	(0.047)	(0.053)	(0.039)
Control variables	Control	Control	Control
Time effect	Yes	Yes	Yes
Individual effect	Yes	Yes	Yes
Constant	−0.120	−0.179	−0.131
	(0.281)	(0.242)	(0.452)
Observations	5,152	3,770	3,038
R^2^	0.338	0.294	0.305

The educational human capital in the eastern region has the greatest impact on the risk of relapse into poverty, which may be due to the higher level of economic development in the eastern region, which provides more resources and better conditions for education investment. A high economic level means that the government and the private sector have more funds to invest in education, thus improving the quality of education and increasing the penetration rate and accessibility of education [74]. In the eastern region, the allocation of educational resources is more optimized and can more effectively meet the needs of different families. Families in the eastern region are able to make better use of educational resources and improve the skills and qualities of family members through education, thereby increasing sources of income and reducing the risk of families falling back into poverty. This conclusion is further substantiated by complementary research demonstrating that enhancements in educational quality and expanded access to learning resources correlate with elevated employment opportunities and wage growth among household members.

Geographic and environmental conditions exert profound influence on poverty dynamics, particularly in ecologically vulnerable regions. Southwestern China’s mountainous and high-altitude areas exemplify this phenomenon, where marginalized households confront persistent barriers to sustainable development due to terrain-induced agricultural limitations and climatic constraints [75]. Concurrently, inadequate infrastructure in these regions perpetuates economic stagnation by restricting market access, deterring external investment, and impeding service provision, thereby creating a vicious cycle of underdevelopment that undermines poverty eradication efforts. This infrastructure deficit manifests in diminished quality of life for residents and constrained entrepreneurial opportunities, limiting the efficacy of anti-poverty interventions [3]. Furthermore, the geographical distribution of poverty alleviation resources exhibits marked disparities, influenced by topographical complexity, remote location, and transportation challenges that hinder equitable policy implementation [76].

In China’s central region, comparative economic underdevelopment exacerbates these challenges through diminished returns to human capital investment. With household income levels lagging behind coastal and western areas, low-income families face heightened opportunity costs and uncertain future benefits from educational expenditures, leading to suboptimal investment in higher education [69]. This regional disparity is compounded by systemic deficiencies in educational infrastructure and faculty resources, particularly in rural central regions, where resource allocation imbalances persist. Central Chinese provinces demonstrate significantly lower educational expenditure compared to eastern and western counterparts, directly impairing instructional quality and facility modernization [77]. Empirical evidence confirms that fiscal education allocations in southwest and northwest provinces yield marginal poverty-reduction effects, reflecting chronic underfunding and inadequate growth in educational budgets across these regions [23]. Such multidimensional vulnerabilities necessitate integrated policy frameworks that address geographic disadvantages while enhancing educational equity and economic resilience in historically marginalized areas.

## 6. Discussion

### 6.1 Study overview and unique contributions

Utilizing longitudinal data from the CFPS spanning 2014–2020, this study empirically examines the impact and transmission pathways of educational human capital on the risk of relapse into poverty in households. The findings confirm that educational human capital significantly reduces the risk of relapse into poverty by facilitating non-agricultural employment, increasing household income, and enhancing the health status of family members. It is important to note that the analysis reveals a nuanced dynamic: while educational human capital acts as a protective buffer, the financial burden associated with health spending can undermine this inhibitory effect.

Consistent with prior research, this analysis validates the critical role of educational attainment in preventing the recurrence of poverty [78–80]. However, this paper focuses on the impact of educational human capital on the risk of relapse into poverty and further demonstrates that educational human capital can significantly reduce the risk of relapse into poverty. Compared with existing studies, the contribution of this paper lies not only in reaffirming the poverty reduction function of education, but also in extending the analytical perspective from poverty alleviation outcomes to poverty reduction sustainability. In this sense, this study speaks more directly to the emerging literature on vulnerability, resilience and long-term stability in poverty alleviation.

This paper distinguishes itself from the existing literature in three key respects. First, unlike prior studies that focus primarily on static poverty alleviation, this paper addresses the dynamic risk of poverty relapse, addressing a critical gap in sustainable development research. This shift in perspective is important theoretically because it shifts the focus from whether households escape poverty at one point in time to whether they have sufficient capacity to withstand future shocks. Educational human capital, in this framework, should be understood not merely as an income-enhancing asset, but as a resilience-enhancing capability that strengthens the long-term development potential of households.

Second, the study systematically elucidates multiple indirect mechanisms. Specifically, it identifies labor market transitions, income uplift, and health improvements. Through these pathways, education fortifies household resilience. This finding enriches the theoretical understanding of how education operates in the anti-poverty process. Rather than exerting influence through a single direct channel, educational human capital appears to influence poverty relapse risk through a multidimensional process of capacity enhancement, in line with the broader human capital and capacity development literature.

Third, the paper explores the regional and urban-rural heterogeneity of these impacts. This analysis provides a granular evidence base. It supports precision decision making, providing a clear improvement over the broad-brush approach found in prior literature. From an empirical point of view, the heterogeneity analysis further suggests that the poverty prevention effect of education is embedded in the local opportunity structure. In other words, the return on educational human capital depends not only on household endowments themselves, but also on regional labour markets, public services and institutional conditions capable of translating educational accumulation into stable welfare gains.

### 6.2 In-depth discussion of findings

Our empirical analysis yields insights into the mechanisms of poverty prevention.

First, non-agricultural employment serves as a primary channel. Higher educational attainment enables rural laborers to transition into the industrial and service sectors, diversifying income sources beyond volatile agricultural earnings [78]. This shift reduces reliance on a single income stream, thereby buffering families against economic fluctuations [79]. This result is highly consistent with the structural transformation literature, which argues that education enhances labour mobility and improves the ability of individuals to enter sectors with higher productivity and more stable returns. In the context of poverty prevention, the significance of this mechanism lies not only in higher wages, but also in reduced vulnerability associated with a more diversified and less climate-dependent livelihood.

Second, education mitigates the risk of relapse through income uplift. By augmenting individual earning capacity, educational attainment directly raises household income levels, particularly in low-income contexts where upward mobility is essential for stability [80,81]. This finding is consistent with the classical human capital proposition that education increases productivity and earnings. More importantly, our results suggest that the income effect of education should be understood dynamically: higher and more stable incomes not only help households cross the poverty line, but also improve their capacity to absorb adverse shocks, smooth consumption, and maintain investment in productive activities.

Third, education functions through a health improvement mechanism. It enhances health behaviors and awareness, leading to higher labor productivity and preventing poverty caused by illness [82]. Considering mitigating factors, the analysis highlights that household investment in education amplifies the mitigating effect of human capital. Such investments facilitate intergenerational mobility and long-term economic stability [83,84]. Household health care spending, in turn, significantly undermines this protective effect. In low-income households, high health costs crowd out funds for education and other long-term investments, weakening the stability of poverty alleviation [85]. This mechanism also resonates with the literature that emphasizes the complementarity between education and health. Better-educated household members are more likely to adopt preventive health behaviors, make more rational medical decisions and use health information more effectively. These advantages can reduce both the direct health risks and the indirect economic losses caused by illness, thereby reducing the probability of return to poverty.

Finally, the heterogeneity analysis confirms the spatially differentiated effect. While protective effects are observed across the country, eastern and urban areas derive significantly greater benefits from education investments compared to central/western and rural areas, reflecting a systemic gap in resource allocation. This pattern suggests that educational human capital does not generate the same returns across different contexts. Regions with more developed markets, better infrastructure and stronger public service provision are better able to translate education into jobs, higher earnings and stronger social protection. In contrast, in less developed or more rural areas, the poverty prevention impact of education may be constrained by limited labor market absorption, weaker institutional support, and fewer complementary resources. This also helps explain why similar levels of educational attainment may produce unequal welfare outcomes across regions.

## 7. Policy implications and limitations

### 7.1 Policy implications

Based on these findings, we propose the following targeted policy interventions to sustain poverty alleviation outcomes.

First, prioritizing educational empowerment for vulnerable groups. Given the robust inhibitory effect of education on the relapse of poverty, policymakers should continue to prioritize the allocation of educational resources in previously impoverished areas. Strategies should move beyond basic access to focus on the quality of education and vocational training, particularly for households identified as high risk, to maximize the marginal benefits of human capital. At the same time, the state should provide demand-driven job training. Courses tailored to market needs, such as e-commerce, will align rural skills with industry demands. Moreover, the authorities should incentivize corporate participation. Offering tax breaks and subsidies to companies that recruit rural workers would structurally expand the supply of stable jobs. These measures can help transform educational accumulation into sustainable employability and enhance the resilience of vulnerable households against future poverty shocks.

Second, policies must strengthen the hematopoietic capacity of households through financial and educational support. To ensure that income uplift translates into resilience, the government should deepen educational assistance. Expanded precision-targeted scholarships based on poverty level can ensure equitable access to schooling. In addition, comprehensive entrepreneurship programs are essential. The authorities should provide training, preferential site rentals and inclusive microfinance with lower interest rates. These measures empower households to use their human capital for business creation. Moreover, the state should incentivize household educational investment through tax reductions and tiered subsidies. Community organizations should also conduct workshops to guide parents in strategic educational planning. In this way, public support can reinforce both short-term income generation and long-term intergenerational mobility.

Third, social protection is integrated with human capital development. The finding that health spending dampens the protective effect of education highlights the need for a multi-layered social safety net. Strengthening health care security and critical illness insurance is essential to prevent health shocks from eroding the long-term benefits of educational accumulation and thus preventing a relapse into disease-induced poverty. Crucially, authorities must keep a tight rein on health costs. Strengthening price regulations and establishing early warning mechanisms will prevent excessive health spending. This ensures that health costs do not crowd out vital household investments in education. In addition, coordinated policies linking education assistance, medical protection and household welfare monitoring will help form a more integrated prevention mechanism against the relapse of poverty.

Fourth, policymakers should implement geographically targeted interventions based on regional heterogeneity. In the eastern region, the allocation of educational resources must be aligned with strategic emerging industries to cultivate specialized talent. In turn, the central and western regions are demanding accelerated infrastructure development. Initiatives for digital equity and teacher capacity building are critical to bridge the quality gap. Moreover, the urban-rural integration mechanism must be strengthened. Strategies such as teacher rotation systems and rural vocational training networks will enhance educational returns in rural areas. These differentiated approaches ensure inclusive poverty alleviation outcomes across a diverse socioeconomic landscape.

### 7.2 Limitations

There are several limitations to this study. First, its scope is largely limited to basic and vocational education. Higher education, with its capacity for intellectual innovation, and further education, with its targeted flexibility, both have unique potential to mitigate the relapse of poverty. Future research should develop a more comprehensive analytical framework. This would allow for a systematic comparison of how different types of education reduce the risk of relapse into poverty, thereby enriching theoretical understanding of the role of education in poverty alleviation.

Second, the data do not adequately capture the actual risk of return to poverty. Available data sets do not adequately represent the complexity and diversity of this risk across different households. Subsequent studies could employ a case study approach. Using field studies and qualitative surveys to obtain primary data would provide a deeper insight into the internal mechanisms and factors that influence the relapse of household poverty. Analyzing these typical cases can yield valuable lessons to guide prevention efforts in other regions.

Third, although this study uses four waves of CFPS panel data from 2014 to 2020, there are temporal gaps between the survey waves. While this panel structure provides valuable longitudinal information, discontinuous observation intervals may limit our ability to capture short-term fluctuations in household poverty relapse risk and the immediate effects of changes in educational human capital. Some transient shocks, such as sudden health events, temporary employment disruptions, or short-term income losses, may occur between waves and therefore cannot be fully observed in a timely manner. In this sense, the findings of this paper should be interpreted primarily as reflecting medium- to long-term relationships rather than high-frequency dynamical changes. Future survey waves would benefit from more continuous follow-up or shorter observation intervals, which would help improve the accuracy of identifying the timing and process of poverty relapse. For researchers and data users, this also suggests the need for caution when drawing inferences about short-term household welfare dynamics from multi-wave panel data.

Fourth, while the results suggest that non-farm employment, and in particular the higher share of migrant workers within households, is an important channel through which educational human capital reduces the risk of relapse into poverty, the sustainability of this pathway may vary over time. In the context of rural revitalization, policy support has increasingly expanded beyond migrant employment to include home-grown entrepreneurship, local job creation and rural industrial development. These changes may alter the migration patterns underlying the mechanisms identified in this study. In addition, reliance on migration may involve several trade-offs, including reduced local productive capacity under sustained outward migration, greater reliance on remittance income, and unequal access to migration opportunities across households. While these issues are beyond the scope of the present study, they should be considered when interpreting the wider policy implications of the findings. Future research could examine how changing rural labor mobility and local development strategies shape the long-term poverty prevention impact of educational human capital.

Finally, the study did not fully explore the synergistic effects between education and other poverty alleviation factors. Industrial development creates jobs and raises incomes. Social security relieves financial stress. Policy support provides institutional guarantees. Future research should integrate multidisciplinary theories from econometrics and sociology. This would enable a systematic analysis of the collaborative effects between education, industry, social security and policy. Such work would provide a theoretical basis for the formulation of comprehensive anti-poverty strategies. Future research may also combine panel survey data with administrative records, local surveillance data, or qualitative evidence to obtain a more continuous and multidimensional understanding of household vulnerability trajectories. Addressing these issues will enrich theoretical and practical research on the reoccurrence of poverty. It will provide more scientific support for crafting precise and effective policies. Ultimately, this will help consolidate poverty alleviation achievements and facilitate a seamless transition to rural revitalization.

## 8. Conclusions

Following the eradication of absolute poverty, preventing poverty relapse has become a major policy challenge in China’s transition toward rural revitalization. Using CFPS 2014–2022 longitudinal data and a two-way fixed-effects model, this study examined whether and through which channels educational human capital affects household risk of relapse into poverty.

Three main conclusions can be drawn. First, educational human capital significantly reduced the risk of relapse into poverty, and this protective effect was stronger in households at higher risk of relapse. Second, the effect operates primarily through non-farm employment and, to a lesser extent, through higher household incomes and better health. Third, the effect varies across household and regional contexts: household investment in education strengthens the poverty prevention effect of education, whereas heavy health spending weakens it; Moreover, the effect is more pronounced in eastern and urban areas than in central and western areas and rural areas.

These findings contribute to the literature in several ways. Conceptually, they extend the role of education from poverty reduction to poverty prevention by showing that educational human capital helps strengthen household capacity to withstand future shocks. Methodologically, the use of longitudinal data, two-way fixed-effects estimation, mechanism analysis, heterogeneity analysis, and multiple robustness checks increased the confidence of the findings. Empirically, the results suggest that the poverty prevention effect of education depends not only on educational attainment itself, but also on employment opportunities, health burdens, and regional development conditions.

Overall, this study provides longitudinal evidence that educational human capital is an important component of sustainable poverty reduction. Policies that expand access to education, improve the translation of education into stable employment, and alleviate the burden of health-related expenditures may help prevent the relapse of poverty and consolidate poverty alleviation achievements in the context of rural revitalization.

## References

[1] Yang Y, de Sherbinin A, Liu Y. China’s poverty alleviation resettlement: progress, problems and solutions. Habitat Int. 2020;98:102135. doi: 10.1016/j.habitatint.2020.102135

[2] Awan A, Bilgili F, Rahut DB. Energy poverty trends and determinants in Pakistan: empirical evidence from eight waves of HIES 1998–2019. Renew Sustain Energy Rev. 2022;158:112157. doi: 10.1016/j.rser.2022.112157

[3] Guo J, Wang L, Zhou W, Wei C. Powering green digitalization: evidence from 5G network infrastructure in China. Resour Conserv Recycl. 2022;182:106286. doi: 10.1016/j.resconrec.2022.106286

[4] DiPrete TA. The impact of inequality on intergenerational mobility. Annu Rev Sociol. 2020;46(1):379–98. doi: 10.1146/annurev-soc-121919-054814

[5] Chen G, Tang G. Spatial non-integration of secondary and tertiary industries from the perspective of the internet: empirical evidence from the Yangtze River Delta urban agglomeration. China Ind Econ. 2016;(08):76–92.

[6] Bi C, Li S. Does tourism development contribute to the green water-use efficiency of the Yellow River Basin in China? J Environ Manag. 2024;351:119933. doi: 10.1016/j.jenvman.2023.119933 38157573

[7] Chen C, Wang JJ, Wang XJ. Does rural industrial integration promote the green development of agriculture?– based on data from 30 provinces in China from 2010 to 2021. HARD Publishing Company; 2023.

[8] Cai H, Wang Z, Ji Y, Xu L. Digitalization and innovation: How does the digital economy drive technology transfer in China? Econ Model. 2024;136:106758. doi: 10.1016/j.econmod.2024.106758

[9] Carter MR, Barrett CB. The economics of poverty traps and persistent poverty: an asset-based approach. J Dev Stud. 2006;42(2):178–99. doi: 10.1080/00220380500405261

[10] Alkire S, Foster J. Counting and multidimensional poverty measurement. J Public Econ. 2011;95(7–8):476–87. doi: 10.1016/j.jpubeco.2010.11.006

[11] Angulo R, Díaz Y, Pardo R. The Colombian multidimensional poverty index: measuring poverty in a public policy context. Soc Indic Res. 2015;127(1):1–38. doi: 10.1007/s11205-015-0964-z

[12] Antonioli D, Berardino CD, Onesti G. The intersectoral linkages and manufacturing productivity growth in Italian regions using the I-O approach. Struct Change Econ Dyn. 2023;66:120–33. doi: 10.1016/j.strueco.2023.04.007

[13] Babu MM, Rahman M, Alam A, Dey BL. Exploring big data-driven innovation in the manufacturing sector: evidence from UK firms. Ann Oper Res. 2021:1–28. doi: 10.1007/s10479-021-04077-1 33897083 PMC8058601

[14] Alkire S, Santos ME. Measuring acute poverty in the developing world: robustness and scope of the multidimensional poverty index. World Dev. 2014;59:251–74. doi: 10.1016/j.worlddev.2014.01.026

[15] Chakravarty S, Lundberg M, Nikolov P, Zenker J. Vocational training programs and youth labor market outcomes: evidence from Nepal. J Dev Econ. 2019;136:71–110. doi: 10.1016/j.jdeveco.2018.09.002

[16] Halpern-Manners A, Schnabel L, Hernandez EM, Silberg JL, Eaves LJ. The relationship between education and mental health: new evidence from a discordant twin study. Soc Forces. 2016;95(1):107–31. doi: 10.1093/sf/sow035

[17] Psacharopoulos G, Patrinos HA. Returns to investment in education: a decennial review of the global literature. Educ Econ. 2018;26(5):445–58. doi: 10.1080/09645292.2018.1484426

[18] Troger T, Verwiebe R. The role of education for poverty risks revisited: couples, employment and profits from work–family policies. J Eur Soc Policy. 2015;25(3):286–302. doi: 10.1177/0958928715589068

[19] Pritchett L. Where has all the education gone? World Bank Econ Rev. 2001;15(3):367–91. doi: 10.1093/wber/15.3.367

[20] Attanasio O, Kaufmann K. Education choices and returns to schooling: intrahousehold decision making, gender and subjective expectations. J Dev Econ. 2014;109:203–16.

[21] Wan G, Hu X, Liu W. China’s poverty reduction miracle and relative poverty: focusing on the roles of growth and inequality. China Econ Rev. 2021;68:101643. doi: 10.1016/j.chieco.2021.101643

[22] Knight J, Deng Q, Li S. The puzzle of migrant labour shortage and rural labour surplus in China. China Econ Rev. 2011;22(4):585–600. doi: 10.1016/j.chieco.2011.01.006

[23] Jiang Z, Zhang X, Zhao Y, Li C, Wang Z. The impact of urban digital transformation on resource sustainability: evidence from a quasi-natural experiment in China. Resour Policy. 2023;85:103784. doi: 10.1016/j.resourpol.2023.103784

[24] Chen Y, Chen S, Miao J. Does smart city pilot improve urban green economic efficiency: accelerator or inhibitor. Environ Impact Assess Rev. 2024;104:107328. doi: 10.1016/j.eiar.2023.107328

[25] Linxiu Z. Employment, emerging labor markets, and the role of education in rural China. J Comp Econ. 2001;29:505–26.

[26] Sunam R, Barney K, McCarthy JF. Transnational labour migration and livelihoods in rural Asia: tracing patterns of agrarian and forest change. Geoforum. 2021;118:1–13. doi: 10.1016/j.geoforum.2020.11.004

[27] Appio FP, Frattini F, Petruzzelli AM, Neirotti P. Digital transformation and innovation management: a synthesis of existing research and an agenda for future studies. J Prod Innov Manag. 2021;38(1):4–20. doi: 10.1111/jpim.12562

[28] Chatti W, Majeed MT. Information communication technology (ICT), smart urbanization, and environmental quality: evidence from a panel of developing and developed economies. J Clean Prod. 2022;366:132925. doi: 10.1016/j.jclepro.2022.132925

[29] Bai YL, Zhang LX, Sun MX, Xu XB. Status and path of intergenerational transmission of poverty in rural China: a human capital investment perspective. J Integr Agric. 2021;20(04):1080–91.

[30] Gao X, Yan X, Song S, Xu N. The impact and mechanism of new-type urbanization on new quality productive forces: empirical evidence from China. Sustainability. 2025;17(1):353. doi: 10.3390/su17010353

[31] Geng W, Wang X, Wang X. National big data integrated pilot zones and FDI inflow: a quantitative and qualitative perspective. Int Econ Trade Res. 2023;39(01):19–35.

[32] Brunello G, Fort M, Schneeweis N, Winter-Ebmer R. The causal effect of education on health: what is the role of health behaviors? Health Econ. 2016;25(3):314–36. doi: 10.1002/hec.3141 25581162

[33] Hubbard S. Education and financial knowledge in health-related financial decisions. Health Educ Behav. 2024;51(5):710–8. doi: 10.1177/10901981241227168 38258824

[34] Behrman JR, Parker SW, Todd PE. Schooling impacts of conditional cash transfers on young children: evidence from Mexico. Econ Dev Cult Change. 2009;57(3):439–77. doi: 10.1086/596614 20209076 PMC2832207

[35] Caputo F, Cillo V, Candelo E, Liu Y. Innovating through digital revolution: the role of soft skills and big data in increasing firm performance. Manag Decis. 2019;57(08):2032–51.

[36] Carneiro P, Meghir C, Parey M. Maternal education, home environments, and the development of children and adolescents. J Eur Econ Assoc. 2012;11:123–60. doi: 10.1111/j.1542-4774.2012.01096.x

[37] Saksena P, Hsu J, Evans DB. Financial risk protection and universal health coverage: evidence and measurement challenges. PLoS Med. 2014;11(9):e1001701. doi: 10.1371/journal.pmed.1001701 25244520 PMC4171370

[38] Begum A, Hamid SA. Impoverishment impact of out-of-pocket payments for healthcare in rural Bangladesh: Do the regions facing different climate change risks matter? PLoS One. 2021;16(6):e0252706. doi: 10.1371/journal.pone.0252706 34086781 PMC8177643

[39] Piroozi B, Moradi G, Nouri B, Mohamadi Bolbanabad A, Safari H. Catastrophic health expenditure after the implementation of health sector evolution plan: a case study in the west of Iran. Int J Health Policy Manag. 2016;5(7):417–23. doi: 10.15171/ijhpm.2016.31 27694669 PMC4930347

[40] Pritchett L, Suryahadi A, Sumarto S. Quantifying vulnerability to poverty: a proposed measure, applied to Indonesia. No. 2437. World Bank Publications; 2000.

[41] Chaudhuri S, Jalan J, Suryahadi A. Assessing household vulnerability to poverty from cross-sectional data: a methodology and estimates from Indonesia. Columbia University. Discussion Paper; 2002.

[42] Hoddinott J, Quisumbing A. Methods for microeconometric risk and vulnerability assessments. Social Protection Discussion Paper. London: Palgrave Macmillan UK; 2010. p. 62–100.

[43] Chambers R, Conway G. Sustainable rural livelihoods: Practical concepts for the 21st century. IDS Discussion Paper. Brighton: Institute of Development Studies; 1992. 296 p.

[44] Hahn MB, Riederer AM, Foster SO. The Livelihood Vulnerability Index: a pragmatic approach to assessing risks from climate variability and change—A case study in Mozambique. Glob Environ Change. 2009;19(1):74–88. doi: 10.1016/j.gloenvcha.2008.11.002

[45] Briguglio L, Cordina G, Farrugia N, Vella S. Economic vulnerability and resilience: concepts and measurements. In: Measuring vulnerability in developing countries. Routledge; 2014. p. 47–65.

[46] Joint Research Centre. Handbook on constructing composite indicators: methodology and user guide. Paris: OECD Publishing; 2008.

[47] Chambers R, Conway G. Sustainable rural livelihoods: practical concepts for the 21st century. Institute of Development Studies; 1992.

[48] Sen A. Development as freedom. Oxford University Press; 1999.

[49] Deaton A. The analysis of household surveys: a microeconometric approach to development policy. The World Bank; 1997.

[50] Barrett CB, Carter MR. The economics of poverty traps and persistent poverty: empirical and policy implications. J Dev Stud. 2013;49(7):976–90. doi: 10.1080/00220388.2013.785527

[51] Banerjee A, Duflo E. Poor economics: a radical rethinking of the way to fight global poverty. Soc Econ. 2013;35(04):573–87.

[52] Karlan D, Zinman J. Expanding credit access: using randomized supply decisions to estimate the impacts. Rev Financ Stud. 2009;23(1):433–64. doi: 10.1093/rfs/hhp092

[53] Wagstaff A, Lindelow M. Are health shocks different? Evidence from a multishock survey in Laos. Health Econ. 2014;23(6):706–18. doi: 10.1002/hec.2944 23765700

[54] Bloom DE, Canning D. The health and wealth of nations. Science. 2000;287(5456):1207–9. doi: 10.1126/science.287.5456.1207 10712155

[55] Bird K, Chabé-Ferret B, Simons A. Linking human capabilities with livelihood strategies to speed poverty reduction: evidence from Rwanda. World Dev. 2022;151:105728. doi: 10.1016/j.worlddev.2021.105728

[56] He D, Zhang Z, Han M, Kang Y, Gao P. Multi-dimensional boundary effects and regional economic integration: evidence from the Yangtze River Economic Belt. Int Reg Sci Rev. 2022;45(4):472–98. doi: 10.1177/01600176211061831

[57] Deininger K, Jin S, Nagarajan HK. Land reforms, poverty reduction, and economic growth: evidence from India. J Dev Stud. 2009;45(4):496–521. doi: 10.1080/00220380902725670

[58] Guan AP, Liu KX. The impact of rural women’s human capital on family income: an empirical analysis based on poor villages in Gansu Province. Popul Dev. 2018;24(04):37–47.

[59] Chen Y, Pan X, Liu P, Vanhaverbeke W. How does digital transformation empower knowledge creation? Evidence from Chinese manufacturing enterprises. J Innov Knowl. 2024;9(2):100481. doi: 10.1016/j.jik.2024.100481

[60] Meinzen-Dick R, Quisumbing A, Doss C, Theis S. Women’s land rights as a pathway to poverty reduction: framework and review of available evidence. Agric Syst. 2019;172:72–82. doi: 10.1016/j.agsy.2017.10.009

[61] Zheng XT, Lu XH. Is it good news for women that there are brothers? ——Research on gender discrimination in family human capital investment. Economics (Quarterly). 2018;17(01):277–98.

[62] Chen K, Li Q, Shoaib M, Ameer W, Jiang T. Does improved digital governance in government promote natural resource management? Quasi-natural experiments based on smart city pilots. Resour Policy. 2024;90:104721. doi: 10.1016/j.resourpol.2024.104721

[63] Namazova N. Changing the level of education and career choice depending on the socioeconomic status of the family: evidence from Azerbaijan. Sustainability. 2023;15(22):15845. doi: 10.3390/su152215845

[64] Brown P, James D. Educational expansion, poverty reduction and social mobility: reframing the debate. Int J Educ Res. 2020;100:101537. doi: 10.1016/j.ijer.2020.101537

[65] Karimli L, Ssewamala FM, Neilands TB, Wells CR, Bermudez LG. Poverty, economic strengthening, and mental health among AIDS orphaned children in Uganda: mediation model in a randomized clinical trial. Soc Sci Med. 2019;228:17–24. doi: 10.1016/j.socscimed.2019.03.003 30870668 PMC6502261

[66] Ridley M, Rao G, Schilbach F, Patel V. Poverty, depression, and anxiety: causal evidence and mechanisms. Science. 2020;370(6522):eaay0214. doi: 10.1126/science.aay0214 33303583

[67] Blandin A, Herrington C. Family heterogeneity, human capital investment, and college attainment. Am Econ J: Macroecon. 2022;14(4):438–78. doi: 10.1257/mac.20200014

[68] Engzell P, Tropf FC. Heritability of education rises with intergenerational mobility. Proc Natl Acad Sci U S A. 2019;116(51):25386–8. doi: 10.1073/pnas.1912998116 31792187 PMC6926022

[69] Deng Q, Zhang L. Spatial and temporal evolution of green efficiency of water resources in the Yangtze River Economic Belt and its influencing factors. Resour Sci. 2022;44(02):247–60.

[70] Kolasa A, Weychert E. The causal effect of catastrophic health expenditure on poverty in Poland. Eur J Health Econ. 2024;25(2):193–206. doi: 10.1007/s10198-023-01579-6 36897432 PMC9999341

[71] Mitra S, Palmer M, Mont D, Groce N. Can households cope with health shocks in Vietnam? Health Econ. 2016;25(07):888–907. 26017577 10.1002/hec.3196 PMC4975721

[72] Wang Y, Duan X, Wang L, Zou H. Spatial temporal patterns and driving factors of industrial pollution and structures in the Yangtze River Economic Belt. Chemosphere. 2022;303(Pt 2):134996. doi: 10.1016/j.chemosphere.2022.134996 35597462

[73] Xu T, Chen L, Liang S. Spatial inequality of carbon emissions at the city level in China. J Clean Prod. 2019;208:638–48.

[74] Fu X, Guo C. Digital inclusive finance and the construction of agricultural modernization. Int Rev Econ Finance. 2025;102:104332. doi: 10.1016/j.iref.2025.104332

[75] Dong Y, Jin G, Deng X, Wu F. Multidimensional measurement of poverty and its spatio-temporal dynamics in China from the perspective of development geography. J Geogr Sci. 2021;31(1):130–48. doi: 10.1007/s11442-021-1836-x

[76] Feng Z, Wang X. Does industrial upgrading promote carbon emission reduction? Evidence from China’s manufacturing industry. J Clean Prod. 2020;276:123313.

[77] Ji R, Wang C, Wang P, Wang W, Chen N. Quantitative analysis of spatiotemporal disparity of urban water use efficiency and its driving factors in the Yangtze River Economic Belt, China. J Hydrol: Reg Stud. 2024;51:101647. doi: 10.1016/j.ejrh.2023.101647

[78] Mansi E, Hysa E, Panait M, Voica MC. Poverty—A challenge for economic development? Evidences from Western Balkan countries and the European Union. Sustainability. 2020;12(18):7754. doi: 10.3390/su12187754

[79] Ariken M, Zhang F, Chan N, Kung H. Coupling coordination analysis and spatio-temporal heterogeneity between urbanization and eco-environment along the Silk Road Economic Belt in China. Ecol Indic. 2021;121:107014. doi: 10.1016/j.ecolind.2020.107014

[80] Ge K, Wang Y, Liu X, Lu X, Ke S. Spatio-temporal differences and convergence mechanisms of green transition of urban land use against the background of industrial integration: a case study of the Yangtze River Economic Belt in China. Ecol Indic. 2024;159:111727. doi: 10.1016/j.ecolind.2024.111727

[81] Sabol TJ, Sommer TE, Chase-Lansdale PL, Brooks-Gunn J. Intergenerational economic mobility for low-income parents and their children: a dual developmental science framework. Annu Rev Psychol. 2021;72:265–92. doi: 10.1146/annurev-psych-010419-051001 32966174

[82] Stephens M Jr, Toohey D. The impact of health on labor market outcomes: evidence from a large-scale health experiment. Am Econ J: Appl Econ. 2022;14(3):367–99. doi: 10.1257/app.20180686

[83] Hastings OP, Schneider D. Family structure and inequalities in parents’ financial investments in children. J Marriage Fam. 2020;83(3):717–36. doi: 10.1111/jomf.12741

[84] Wassink JT, Viera JA. Does parental migration during childhood affect children’s lifetime educational attainment? Evidence from Mexico. Demography. 2021;58(5):1765–92. doi: 10.1215/00703370-9411336 34477826

[85] Certomà C. Future scenarios of Digital Social Innovation in urban governance. A collective discussion on the socio-political implications in Ghent. Cities. 2022;122:103542. doi: 10.1016/j.cities.2021.103542

